# Stakeholder perceptions of the acceptability of an intervention to improve uptake of evidence-based emergency myocardial infarction care in Tanzania: A qualitative study

**DOI:** 10.1371/journal.pone.0357564

**Published:** 2026-09-25

**Authors:** Spencer F. Sumner, Francis M. Sakita, Kelvin F. Haukila, Lisa Wanda, Godfrey L. Kweka, Jerome J. Mlangi, Pankrasi Shayo, Tumsifu G. Tarimo, Simran Khanna, Claire Wang, Abigail Pyne, Preeti Manavalan, Nathan M. Thielman, Janet P. Bettger, Julian T. Hertz

**Affiliations:** 1 Duke University School of Medicine, Durham, North Carolina, United States of America; 2 Department of Emergency Medicine, Kilimanjaro Christian Medical Centre, Moshi, Tanzania; 3 Department of Medicine, University of Florida College of Medicine, Gainesville, Florida, United States of America; 4 Duke Global Health Institute, Duke University, Durham, North Carolina, United States of America; 5 Division of Infectious Diseases, Department of Medicine, Duke University Medical Center, Durham, North Carolina, United States of America; 6 Department of Physical Medicine & Rehabilitation, University of North Carolina School of Medicine, Chapel Hill, North Carolina, United States of America; 7 Department of Emergency Medicine, Duke University, Durham, North Carolina, United States of America; Indiana University South Bend, UNITED STATES OF AMERICA

## Abstract

**Background:**

Acute myocardial infarction (AMI) is an increasing cause of morbidity and mortality in Sub-Saharan Africa (SSA) but is often underdiagnosed and undertreated. To address this gap, the Multicomponent Intervention to Improve Myocardial Infarction Care (MIMIC) was developed and implemented to improve evidence-based AMI care. The aim of this study was to explore stakeholder perceptions of the acceptability of MIMIC following its implementation.

**Methods:**

This qualitative study involved in-depth interviews with 20 key stakeholders (physicians, nurses, administrators, and patients diagnosed with AMI) who participated in MIMIC during the first year of implementation in the emergency department (ED) of a regional referral center in northern Tanzania. Purposive sampling was used to recruit diverse participants. Interviews were guided by a semi-structured interview guide informed by the Theoretical Framework of Acceptability (TFA). Interview transcripts were thematically analyzed by a team of coders using an inductive, grounded theory approach guided by the seven TFA domains.

**Results:**

Nineteen major themes emerged across all TFA domains. Overall, participants described MIMIC as acceptable, minimally burdensome, and well-aligned with professional and ethical values. Perceived effectiveness was most emphasized, with staff citing improvements in AMI recognition, electrocardiogram (ECG) and troponin testing, and use of evidence-based therapies. Most components were described as effective and easily integrated into existing workflows. Patients valued the educational pamphlet for improving knowledge and self-efficacy, though staff expressed concerns about distributing it during acute care, contributing to inconsistent delivery. Champions were viewed as key in promoting adherence and sustaining implementation.

**Conclusion:**

MIMIC was found to be acceptable in all seven TFA domains among ED providers and patients, with perceived effectiveness driving positive attitudes across stakeholder groups. MIMIC’s co-design approach likely contributed to high intervention acceptability. Patient education strategies may require adaptation to improve fidelity. These findings support continued implementation and targeted adaptation of MIMIC.

## Introduction

The leading cause of death globally is ischemic heart disease [1]. The incidence of acute myocardial infarction (AMI) is increasing in Sub-Saharan Africa (SSA), [2] where a growing body of evidence has highlighted opportunities to improve AMI care [3]. In northern Tanzania, prior work by our group has shown that AMI is frequently misdiagnosed and inadequately treated, driven by factors including inadequate provider training, insufficient diagnostic resources, and inefficient emergency department systems [4,5]. These gaps in evidence-based AMI care are associated with high mortality, with 30-day mortality exceeding 40% in some reports from the region, compared to single-digit mortality rates in high-income countries [6,7]. Poor AMI outcomes in low-resource settings are further compounded by delayed symptom recognition, delays in seeking care, and barriers to healthcare access—which shape the pathways through which patients reach emergency services [8,9]. Nevertheless, among patients who do present for emergency care, important opportunities remain to improve AMI recognition and treatment through interventions targeting provider knowledge, diagnostic evaluation, and evidence-based management.

In an effort to address these concerns, a multicomponent intervention to improve myocardial infarction care (MIMIC) was implemented at an emergency department in Tanzania [10,11]. MIMIC was developed by an international, interdisciplinary team composed of implementation scientists, physicians, nurses, patients, and social scientists. This intervention consisted of five main components: (a) provider training on AMI recognition, diagnosis, and treatment, (b) triage cards used to identify patients with symptoms concerning for AMI and prompt diagnostic evaluation, (c) provider pocket cards containing concise AMI diagnosis and management algorithms, (d) appointed physician and nursing champions responsible for supporting intervention implementation and addressing barriers to uptake, and (e) patient education delivered through physical and digital pamphlets designed to improve understanding of AMI and its management. Beginning in 2023, the MIMIC intervention was implemented in a pilot trial in the emergency department (ED) at Kilimanjaro Christian Medical Centre (KCMC), in northern Tanzania, to improve uptake of evidence-based AMI care (ClinicalTrials.gov Identifier: NCT04563546) [11].

Preliminary evidence from this pilot trial found that implementation of MIMIC was associated with significant improvements in AMI diagnostics, detection, treatment, and uptake of evidence-based secondary preventative therapies [12]. As with all quality improvement interventions, it is important to determine if the intervention was successful not only through efficacy outcomes, but also through acceptability measures. A previous survey of ED staff using the Acceptability of Intervention Measurement (AIM) and Feasibility of Intervention Measurement (FIM) found that the MIMIC intervention was highly acceptable and feasible from the perspective of ED providers [13,14]. A subsequent follow-up study among KCMC ED clinicians found high perceived organizational capacity for sustainability and strong normalization of MIMIC into routine clinical practice, measured using the Clinical Sustainability Assessment Tool (CSAT) and the NoMAD questionnaire [15]. While these quantitative measures provide important insight into implementation outcomes, they do not fully characterize how stakeholders experienced the intervention or which factors shaped acceptability in practice. Therefore, we used qualitative methods to examine the acceptability of MIMIC among ED physicians, nurses, administrators, and patients to identify factors influencing intervention acceptability. We conducted in-depth interviews with key stakeholders working in, and receiving care from, the KCMC ED during implementation.

## Materials and Methods

### Study design

This study employed a qualitative research design to assess the acceptability and feasibility of the MIMIC intervention in the emergency department (ED) at Kilimanjaro Christian Medical Centre (KCMC) [12]. Semi-structured interviews were conducted with key stakeholders over the one-year study period to explore their perceptions, experiences, and attitudes toward the intervention. The full MIMIC intervention, including triage cards, pocket cards, and educational pamphlets has been previously published [10].

### Setting

KCMC is the Zonal Referral Hospital for northern Tanzania, functioning as a major tertiary care and teaching hospital serving a patient population of roughly 11 million people. The MIMIC intervention was implemented in the KCMC ED beginning on September 1^st^, 2023, and interviews with key stakeholders were conducted from January through September 2024. At the time of the interviews, KCMC lacked a cardiologist on staff and did not have capacity for percutaneous coronary intervention or coronary surgery. AMI patients requiring such care are typically transferred to the national cardiac center in Dar es Salaam. However, as a major referral hospital for northern Tanzania, KCMC routinely serves as the initial point of evaluation and management for patients with suspected AMI prior to transfer. As a result, AMI care at KCMC relies primarily on timely diagnosis and evidence-based medical management, making it an appropriate setting in which to evaluate the acceptability of interventions targeting these aspects of care.

### Participant Sampling and Recruitment

Purposive sampling was used to recruit participants across four key stakeholder groups: nurses, physicians, administrators, and patients. Eligible participants included physicians, nurses, and administrators who worked in the ED during the study period. Eligible patient participants were adults aged 18 years or older with a confirmed diagnosis of AMI in the KCMC ED during the study period who were able to provide informed consent. Within each stakeholder group, participants were purposively selected to ensure diversity across key characteristics including gender, age, and, for provider participants, job title and seniority, and for participants, time since AMI diagnosis. Individuals were excluded only if they were unable to provide informed consent.

Recruitment was conducted through direct invitations by study personnel. Selected hospital staff were approached face-to-face by research staff during break periods and invited to participate, whereas selected patients were contacted by telephone after hospital discharge. Informed consent was obtained from all participants before data collection. All in-depth interview participants received 5,000 Tanzanian shillings (approximately 2 USD) as compensation for their time.

### Data collection

Interviews were guided by a semi-structured interview guide, which was developed by the international, interdisciplinary investigator team (S1 Text). These interview guides were informed by the Theoretical Framework of Acceptability (TFA), which encompasses seven domains of acceptability: affective attitude, burden, perceived effectiveness, ethicality, intervention coherence, opportunity costs, and self-efficacy [16]. The guide included open-ended questions exploring participants’ awareness and understanding of the intervention; perceived benefits and challenges; attitudes towards implementation and sustainability; and barriers and facilitators to intervention uptake. Interviews were conducted face-to-face in Swahili, in a private space, by Tanzanian research assistants with prior qualitative research experience who were not involved in participants’ clinical care. Several members of the research team had pre-existing relationships with KCMC ED staff participants, given the ongoing collaborative partnership between Duke University and KCMC through which the MIMIC intervention was developed and implemented. In the interest of transparency, and to minimize the potential influence of these relationships on participant responses, interviews were conducted by Tanzanian research assistants who had no prior supervisory, evaluative, or clinical relationship with any study participant. Patient participants were recruited solely on the basis of having received an AMI diagnosis in the KCMC ED during the study period, and no members of the research team had pre-existing relationships with any patient participant. Interviewer training covered qualitative interviewing techniques and study-specific protocol review. Each interview lasted approximately 45–60 minutes, and interviews continued until the study team determined thematic saturation had been reached. Repeat interviews were not conducted. All interviews were audio-recorded with participant consent, then transcribed and translated into English. Interviews were initially conducted with 20 participants (n = 5 per stakeholder group). An interim thematic analysis was conducted by three members of the research team (JTH, TGT, SFS), who determined that thematic saturation had been achieved for each stakeholder group, so no additional interviews were conducted.

### Data analysis

The qualitative data analysis for this study was conducted using an iterative, stepwise approach following the thematic framework described by *Applied Thematic Analysis* [17]. The study utilized an inductive approach to identify emergent themes related to intervention acceptability, informed by the TFA. Interview transcripts were coded by trained research personnel with prior experience in qualitative analysis. Coders underwent additional training in thematic analysis principles prior to beginning formal analysis, and the coding team consisted of both cultural insiders (KFH, GLK, LW, JJM, PPS) and cultural outsiders (SS, JTH).

To ensure a rigorous and systematic approach, the coding process began with coders familiarizing themselves with a representative sample of interview transcripts. This initial phase allowed for an in-depth understanding of the interview format and content, ensuring alignment with the research objectives. Following this familiarization, a lead coder developed an initial codebook, informed by the domains of the TFA, that systematically categorized themes of acceptability into discrete codes. These codes identified the acceptability domain, whether a response indicated acceptability or unacceptability, and the specific reasoning underlying the participant’s perspective. The codebook was iteratively refined throughout the coding process as emergent themes were identified through regular discussions between coders. Regular meetings of the study team were also held throughout the analysis process to review coding decisions, refine the codebook, and ensure consistent application of codes across transcripts. All transcripts were independently coded by two members of the coding team, and coding discrepancies were reviewed and reconciled through group discussion and consensus.

Interviews were coded by participant group to ensure that emerging themes could be captured within each stakeholder perspective. The codebook was expanded as necessary with each group, and modifications were made to reflect new themes. Each coder analyzed the interviews independently before convening to discuss discrepancies and reach a consensus. This process was repeated for all four stakeholder groups: nurses, administrators, physicians, and patients. The final codebook is provided in the Supporting Information (S1 Text).

After a consensus was reached for all interviews, thematic analysis was conducted in NVivo 14. High-level thematic analysis was led by two members of the research team (JTH and SS). Dominant themes were identified through iterative review of coded transcripts within each participant group, with consideration given to both the prevalence of themes across interviews and their relevance to understanding intervention acceptability. The seven TFA domains served as the superordinate organizing framework, with the 19 identified themes nested within them; this two-level structure captured the depth of the data without warranting further division into sub-themes. The final stage of analysis involved thematic synthesis, where patterns were identified across stakeholder groups to generate a comprehensive understanding of the intervention’s acceptability within the emergency department setting at KCMC.

Credibility was supported through prolonged engagement with participants, with interviews lasting approximately 45–60 minutes, and through data source triangulation achieved by including four distinct stakeholder groups (physicians, nurses, administrators, and patients) whose perspectives were systematically cross-checked to identify convergent and divergent themes. Dependability was ensured through regular team meetings to review coding decisions and ensure consistent application of codes across transcripts. Confirmability was strengthened through the inclusion of both cultural insiders (KFH, GLK, LW, JJM, PPS) and cultural outsiders (SS, JTH) on the coding team, and through independent dual coding with consensus reconciliation. Transferability was supported through the description of the study context, including detailed characterization of the KCMC ED setting, the MIMIC intervention components, the participant recruitment process, and the sociodemographic characteristics of study participants, enabling readers to assess the applicability of findings to other emergency department settings in Tanzania and similar resource-limited contexts in sub-Saharan Africa.

### Reporting standards

This study is reported in accordance with the Consolidated Criteria for Reporting Qualitative Research (COREQ) checklist (S1 Checklist).

### Ethical considerations

Ethical approval was obtained from the Duke Health Institutional Review Board, the Kilimanjaro Christian Medical Centre Research Ethics Committee, and the Tanzania National Institutes for Medical Research Ethics Coordinating Committee. Written informed consent was obtained from all participants prior to their involvement. Audio recordings were made with participant consent, transcribed verbatim, and translated into English. All transcripts were de-identified prior to analysis through the removal of personally identifiable information. Audio recordings, transcripts, and all other study data were stored securely on password-protected institutional systems accessible only to authorized study personnel. Data management and retention procedures were conducted in accordance with the requirements of all approving institutional review boards and ethics committees. Study data will be stored on a secure server at KCMC for 10 years, as per Tanzania National Institute for Medical Research requirements.

## Results

Twenty participants were enrolled in this study, including five physicians, five nurses, five administrators, and five patients (Table 1). The median age was 32 years (range 27–64), and 40% of participants were female. All five patient participants were literate. Additional participant characteristics are presented in Table 1.

**Table 1:** Participant characteristics, by stakeholder group (n = 20).

	Age: Median (years)	Age: Range	Male (n)	Female (n)
All participants (N = 20)	32	27-64	12	8
Administrators (N = 5)	35	30-49	1	4
Nurses (N = 5)	31	27-32	4	1
Patients (N = 5)	46	32-64	4	1
Physicians (N = 5)	30	28-35	3	2

Across stakeholder groups, we identified 19 dominant themes within all seven TFA domains (Table 2). Overall, participants reported positive affective attitudes toward the MIMIC intervention, describing it as minimally burdensome, well aligned with their professional and ethical values, and effective in improving AMI care. Staff highlighted increased awareness and confidence in diagnosing and treating AMI, while patients emphasized the value of the educational pamphlet in understanding and managing their condition. Divergent views emerged regarding the appropriateness of pamphlet distribution in the ED, representing the main area of disagreement across groups.

**Table 2:** Dominant themes related to MIMIC acceptability, organized by Theoretical Framework of Acceptability domain, intervention component, and stakeholder group.

TFA Domain	Theme	MIMIC component	Number of participants reporting theme	Stakeholder type	Stakeholders reporting theme
Affective Attitude	*Positive attitudes toward MIMIC*	Entire intervention	20	Physicians	5
				Nurses	5
				Administrators	5
				Patients	5
Burden	*Minimal implementation effort*	Entire intervention	14	Physicians	5
				Nurses	5
				Administrators	4
				Patients	0
	*Time and connectivity barriers*	Online training module	8	Physicians	2
				Nurses	5
				Administrators	1
				Patients	0
Ethicality	*Alignment with the staff values*	Entire intervention	15	Physicians	5
				Nurses	5
				Administrators	5
				Patients	0
	*Divergent views on ED pamphlet distribution*	Pamphlet	17	Physicians	3
				Nurses	4
				Administrators	5
				Patients	5
	*Clear understanding of MIMIC*	Entire intervention	20	Physicians	5
				Nurses	5
				Administrators	5
				Patients	5
Opportunity Costs	*MIMIC did not compete with clinical responsibilities*	Entire intervention	14	Physicians	5
				Nurses	4
				Administrators	5
				Patients	0
Perceived Effectiveness	*Improved AMI care*	Entire intervention	15	Physicians	5
				Nurses	5
				Administrators	5
				Patients	0
	*Improved provider knowledge*	Entire intervention	15	Physicians	5
				Nurses	5
				Administrators	5
				Patients	0
	*Support for continued implementation*	Entire intervention	18	Physicians	4
				Nurses	5
				Administrators	5
				Patients	4
	*Support for intervention scale-up*	Entire intervention	17	Physicians	5
				Nurses	4
				Administrators	3
				Patients	5
	*Pamphlet as a patient and community education tool*	Pamphlet	12	Physicians	3
				Nurses	4
				Administrators	5
				Patients	0
	*Red triage card as a visual cue*	Triage card	13	Physicians	5
				Nurses	5
				Administrators	3
				Patients	0
	*Provider pocket card as a clinical reminder*	Provider pocket card	12	Physicians	4
				Nurses	4
				Administrators	4
				Patients	0
*Champions facilitated evidence-based AMI care and MIMIC implementation*	Champions	15	Physicians	5
			Nurses	5
			Administrators	5
			Patients	0
	*Online training-enhanced provider competency*	Online training module	10	Physicians	4
				Nurses	2
				Administrators	4
				Patients	0
	*Inconsistent pamphlet distribution*	Pamphlet	13	Physicians	4
				Nurses	4
				Administrators	5
				Patients	0
Self-Efficacy	*Provider empowerment in AMI management*	Entire intervention	11	Physicians	3
				Nurses	4
				Administrators	4
				Patients	0
	*Patient self-management confidence*	Pamphlet	5	Physicians	1
				Nurses	1
				Administrators	0
				Patients	3

### Affective Attitude

#### Positive attitudes toward MIMIC.

All participants across all stakeholder groups expressed positive affective attitudes towards the MIMIC intervention. However, perceptions of individual intervention components varied across stakeholder groups. For example, as discussed below, patients expressed strong positive feelings about the educational pamphlets, while feelings towards the pamphlets among administrators were more mixed. Nonetheless, all participants expressed an overall positive attitude toward MIMIC.

“Yeah. I would frown filling in a death certificate but not MIMIC.”

[…]

I have never seen a frown face when it comes to MIMIC.” (Physician, M)

“I liked MIMIC. Not only liked it but I loved it! And I still love it!

[…] Everyone that I met thinks it is a very good intervention.” (Physician, F)

Patients who only engaged with the educational pamphlet component of MIMIC also reported uniformly positive feelings towards this intervention.

I: “In general, how do you feel about this educational pamphlet?”

R: “The pamphlet is good […]. They gave me that pamphlet, it helped me, and I received it because I knew that I already had MI and what was required for me to do, and at what time.” (Patient, M)

### Burden

#### Minimal implementation effort.

Participants generally viewed MIMIC as minimally burdensome to implement because improving quality of care was considered to be a core professional responsibility. The intervention was perceived as simple, easy to understand, and requiring little additional time or effort.

Moreover, providers felt that MIMIC, which was co-designed by KCMC ED staff and tailored to the Tanzanian context, fit within existing workflows. For example, participants cited the way in which the “MI Suspect” red triage cards could be integrated seamlessly into the existing card-based triage system without creating additional tasks for staff. Furthermore, as MIMIC became normalized, participants reported that it became part of routine ED operations.

“I really don’t see the burden because it is not something completely new. We are using triage cards that we also had before. We have other cards that show if the patient has been seen, if they are awaiting responses and so on. Doing ECG and other tests are part of the doctor’s consultation. Doing troponin in the ED has also made things easier for us. Pocket cards do not give the doctors a burden but rather reminds them on what they are supposed to do. At the emergency we also have charts that remind us on different other diseases so it’s just similar. I don’t think they are a burden I think they are just part of normal routine.” (Administrator, F)

“MIMIC is not a hard thing to do. It is not burdensome. It doesn’t demand a lot from you, you just need to remember a few things. Do this, do that, remember the red tag and follow the protocol. Just a few things that don’t consume your time and energy.” (Physician, F)

#### Time and connectivity barriers.

Although participants felt that MIMIC was minimally burdensome overall, some considered the online module somewhat burdensome to complete. A concern shared by nurses, administrators, and doctors was that an additional online training course was a burden because a busy clinical schedule necessitated the course to be completed on one’s own time outside of the hospital. Because no protected work time was allocated, providers often completed the course at home using a personal internet bundle, which raised concerns about cost and internet access. Although no participant reported personally being unable to access internet to complete the module, many expressed concern that limited internet access or smartphone availability could pose barriers for other staff.

“This is dependent on the overlapping of tasks. You find that you have had a busy day at work, and you have been asked to do an online module. When you get home, you are exhausted, and all you want to do is just rest and say that you will do it on the next day. And that is the beginning of procrastination. Sometimes you cannot do it because of data and internet issues. When you ask some people to do it, they tell you that they do not have data and internet, or their phones are faulty. Those are some of the barriers” (Nurse, M)

### Ethicality

#### Alignment with staff values.

All groups interviewed believed that the MIMIC intervention aligned with both hospital and professional values. When discussing hospital values, participants emphasized that as a tertiary care center, KCMC has a responsibility to provide evidence-based AMI care, and MIMIC helped achieve this goal. In terms of professional ethics, both nurses and physicians noted that MIMIC enhanced their core responsibility to save lives.

“I: Do you think the MIMIC intervention aligns with your values?

R: Yes, it does completely because our aim in providing care is to get quality care. How is quality care described? Have we been able to follow the requirements to get quality care? So, I see it aligns with my values.” (Administrator, F)

#### Divergent views on ED pamphlet distribution.

Providers and patients had notably different perspectives on the appropriateness of distributing educational pamphlets to patients in the ED. Many providers felt that patients with suspected AMI were too acutely ill and distressed to benefit from written educational materials. Instead, they advocated for pamphlet distribution later in the hospitalization or in community settings such as markets, churches, or mosques. This sentiment was particularly common among departmental administrators. Providers also noted that ED doctors were focused on delivering life-saving care, which limited their ability to distribute pamphlets consistently. These participants suggested pamphlet distribution in lower-acuity settings such as inpatient wards or outpatient clinics.

“You know in the case of emergencies, relatives or the patients are mostly in shock and unsettled. If you give them something to read, they keep it in their bags, they don’t read it. That’s my observation.” (Administrator, F)

“As I mentioned earlier, most patients that come here are very old, very sick, in pain and uncooperative. So, we need to improve because we need to deal with the society that is caring for that patient and the population of society that is healthy, so that when they get that education, they may bring their patients early before they reach the end stage of AMI. Also, when the healthy society is educated, they will know how to prevent and control AMI. Not wait until when they reach that [end] stage” (Administrator, F)

In contrast, patients expressed uniformly positive views regarding the timing and location of pamphlet distribution. Several described the pamphlet as an important educational tool, stating that they helped them understand their disease and contributed to a sense of self-efficacy. Many referenced inadequate doctor-patient communication throughout their hospital stay and felt that the pamphlets helped fill gaps in the information they received.

“[receiving the pamphlet in the hospital] matched my value because I received that pamphlet because I was diagnosed of a certain disease. After realizing that I had a certain a problem, the doctor explained it to me, I received the news and understood. Afterwards, he gave me that pamphlet which was like an addition to what he had already explained to me. I received the news verbally and in written form and it is better to have it in writing because you can keep that.” (Patient, M)

“[Providing the pamphlet] is important. It is helpful, it gives people awareness regarding cardiovascular issues. I have been very sick and since I noted it as a problem, I have been hearing from people with MI that they are experiencing challenges like difficulty in breathing, or they have been hospitalized. It is very important and would be helpful if they continued providing them.” (Patient, M)

### Intervention Coherence

#### Clear understanding of MIMIC.

Participants consistently demonstrated a clear understanding of MIMIC and its individual components, with most able to describe the intervention and its purpose without prompting. They understood both the overall goal of improving AMI diagnosis and treatment, as well as the role of MIMIC in achieving this goal. Several participants also noted their involvement in MIMIC’s participatory co-design process. Both the patient pamphlet and provider pocket card were described as clear and easy to understand by both patients and providers because of their simple language and use of Swahili.

“MIMIC is an intervention that helps us identify and manage patients with myocardial infarction. Mostly identification of patients is done once they come into the ED with a red card, so we stay alert that this patient is a suspected case of MI. We also did an online course that helped us gain knowledge on myocardial infarction. We were also given pocket cards which reminded us of the management and diagnosis of myocardial infarction. Most of us have them in our pockets and phones. We also have patient education pamphlets that we give to the patients’ relatives which help shorten the explanation that you need to give to the patient or their relatives. After giving out those pamphlets, the doctor’s job is just to clarify on the things that they did not understand from the pamphlet. This will help increase patients’ knowledge on this disease. Champions also motivate us to ensure that we provide the appropriate management to MI patients and to remind the students and interns on the proper management of MI.

[…]

“I don’t think that it’s a very difficult thing to understand” (Physician, F)

“Honestly, the part that I understood really well is the [Patient education pamphlet] which discusses what MI is and how to prevent it for people without MI. How to avoid MI for example looking at the foods we eat, tobacco use and weight. It has really helped me as an MI patient. If someone asks me what causes MI, it’s easy for me to explain.” (Patient, M)

### Opportunity Costs

#### MIMIC did not compete with clinical responsibilities.

Participants stated that MIMIC did not detract from other important clinical tasks. Because AMI was viewed as life-threatening, its diagnosis and management were viewed as the highest clinical priority. Participants also emphasized that implementing MIMIC was simply a part of their responsibility to provide high-quality care and required minimal additional time or effort.

“So, it can`t distract me from important work, there is nothing that is as important to me, as the patients during my shift. So as long as I am at the emergency department and patient comes in with acute coronary syndrome, I have to go through and adhere to MIMIC. It’s not a distraction, it just draws attention.” (Physician, F)

“I: Have you ever thought that the MIMIC intervention may distract you from other important work?

R: As I mentioned earlier, it is part of my work, so it doesn’t affect anything. Not only for me but also for my colleagues.” (Administrator, F)

### Perceived Effectiveness

#### Improved AMI care.

Perceived effectiveness was the most frequently discussed domain of MIMIC acceptability among participants. Physicians, nurses, and administrators agreed that the intervention substantially improved AMI care quality in the KCMC ED. Participants noted enhanced uptake of ECG and troponin testing, reduced missed AMI diagnoses, and increased use of evidence-based therapies like aspirin and clopidogrel. Perceived effectiveness was also a major driver of overall intervention acceptability. Staff expressed pride in observable improvements in clinical practice, including an improved ability to rapidly recognize AMI symptoms, greater confidence in using diagnostic tools, and more timely treatment decisions, such as transfers to specialized centers or thrombolytic administration. Several also noted a cultural shift in the ED, with AMI now recognized as a time-sensitive emergency requiring immediate attention, directly contributing to better long-term survival outcomes.

“Honestly, I see that we have gone a step ahead because some patients were mismanaged. People didn’t think it was important to give them Aspirin and Clopidogrel, let’s just give them one. But when you have [MIMIC], you can see. Is it important to do an ECG again? But [MIMIC] reminded us. I think now we are more able to identify the patients with this diagnosis compared to the previous years. […] So, it has given us a wider knowledge on how to manage and exclude other things that limited our management.” (Administrator, F)

“I would say it effectively improved the care because yes, sometimes we miss [AMI cases] but now it’s very very few times. We recognize the cases super-fast and they get referred early and they get saved. Most of the cases.” (Physician, F)

“It is good because it helps in identifying patients with AMI, which accounts for many lives. This problem was there and was hurting many people and many people died for that reason. Patients with AMI were coming to the emergency department and there were formerly many delays including delays in sending the patient to the ward. Now, we have reduced those delays and patients are rushed to [specialty cardiology hospital] with an ambulance and receive treatment on time. Of course, it helps because if MIMIC wasn’t there then we would be missing these diagnoses.” (Nurse, F)

#### Improved provider knowledge.

All physicians, nurses, and administrators described improved AMI knowledge and awareness following MIMIC implementation. Participants attributed these gains primarily to the online training module and provider pocket cards, which reinforced AMI recognition and evidence-based management. Participants thought MIMIC was particularly effective in dispelling common misconceptions, refreshing knowledge among experienced clinicians, and educating new providers.

“I like MIMIC because it increases my awareness on this because initially, I did not know about AMI. It has explained AMI to me, and it still sharpens me. I can even educate community members on this now.” (Nurse, M)

“For me, I am happy because it increases my knowledge. Let’s say I go and work in another hospital where such things have not arrived, I can act as a role model. If someone comes with a chest pain on the left side and it is radiating, I can tell them let’s start with this coming from the few things that I know.”

(Nurse, F)

“I: How about improvement of provider awareness? Do you think MIMIC has been effective at that?

R: I’d personally say yes. If you compare the knowledge I had before this intervention and after, there’s a big gap.” (Physician, F)

#### Support for continued implementation.

Providers strongly supported continued implementation of MIMIC in the KCMC ED beyond the study period’s completion. Staff members described the intervention as essential to improving awareness, recognition, and management of AMI; some even expressed concern that discontinuing the intervention would lead to patient harm. Participants also reported actively maintaining components of MIMIC, as well as introducing new staff to intervention practices.

“Please don’t stop this project, please don’t because we have only done it here at KCMC and we have realized that it is a leading cause of death. If you implement it in all hospitals in Tanzania, you will realize that many people die of myocardial infarction.” (Administrator, F)

“I: Do you think the MIMIC intervention can continue in the KCMC emergency department later on?

R: Absolutely! We have seen that this is a good system. We are celebrating good behavior. We are maintaining it with both hands.” (Administrator, M)

“I would like to thank you for this program because it has made a huge positive impact, something which we wish to continue. As I already mentioned, it shouldn’t end here but we should create an environment for those around us to understand this intervention so as to improve care for all patients.” (Nurse, M)

“The major thing we are thinking about in the MIMIC intervention is for this project to continue because it reminds us on how to care for these patients. If you are a provider and have the ability and skills to care for these patients quickly, it will be a stable carrier full of success. Many of us want this to continue for many years.” (Physician, M)

“It is important. Apart from personally liking the pamphlet, it is important to continue distributing it. It is helpful, it gives people awareness regarding cardiovascular issues. … It is very important and would be helpful if KCMC continued providing them.” (Patient, M)

#### Support for intervention scale-up.

In addition to stating that MIMIC should continue at KCMC, participants expressed a desire to implement the MIMIC intervention at emergency departments and health facilities throughout Tanzania. Many felt that implementation at local district hospitals and community clinics could reduce delays in diagnosis and eliminate the need for referral to larger hospitals, ultimately improving patient outcomes. Some also advocated for broader pamphlet distribution to facilitate community education regarding AMI.

“Many people will be helped. So many! Imagine people travelling from the villages to KCMC! How many hospitals have they skipped? How many hospitals have they been attended? They come here in the late stage. So MIMIC is something very nice, they should not stop. Please continue working on it.” (Administrator, F)

“We are now aware of MI, and we need others to be aware. Tanzania is big, and we wish for patients to be diagnosed as soon as possible if they experience those symptoms. It is very important for this education to be taken to other emergency departments. Not only emergency rooms because some hospitals do not have an emergency department; those are the primary facilities that are attended by many patients who are poor. It should be provided to all facility levels and not only to the big hospitals.” (Physician, F)

“I am happy to be part of this intervention. If where we are planning to go [to other emergency departments] we will be received with open arms and then we will get more patients and we will reach more people.” (Administrator, M)

“You should give pamphlets anywhere patients can be given them. Print out more copies and give the pamphlet to more people. This will help in reducing this problem. People will get to know the symptoms, the type and the numbers [of MI cases] will reduce.” (Patient, M)

#### Pamphlet as a patient and community education tool.

Patients widely described the educational pamphlet as an effective tool for improving understanding of AMI among not only themselves but also their friends, neighbors, and families. Participants reported learning about AMI symptoms, etiologies, prevention, and treatment while also utilizing the pamphlet to teach fellow community members, including those with lower health literacy. Some even described the pamphlet as prompting them to seek emergent care for a loved one with suspected AMI.

“It has improved [things for me] because I didn’t know anything about that disease. When I was given the pamphlet, I read about the symptoms and causes, and I understood that there is a disease of this kind.” (Patient, F)

“The pamphlet is important. It is very important because the more you read it the more you remember. It reminds you the of symptoms, what to do, what to avoid to prevent another heart attack.” (Patient, M)

“Honestly, I read this pamphlet with my children when it was given to me at KCMC. Everybody wanted to know what it was.” (Patient, M)

“All the people that I have shared it with them were eager to read it. Some of them were eager to change because there are some things that they have seen there that cause MI. Some people have seen the need to reduce or stop for instance the smokers. I can see that some are trying to stop. Sometimes they fail but they are trying. The issue of obesity as well because I have spoken to many people and I see that they can understand. People like it, it is nice and some people have partners with this problem, so they understand.” (Patient, M)

#### Red triage card as a visual cue.

The red triage card was identified as a clear visual cue for suspected AMI, immediately prompting ED physicians to consider this diagnosis in their workups. Staff appreciated its visually distinctive appearance, commenting that they could identify the card clearly in a crowded ED and initiate the appropriate diagnostic workup for AMI. Physicians, nurses, and administrators all felt that AMI was commonly overlooked in the KCMC ED prior to MIMIC implementation, primarily due to physicians failing to consider the diagnosis. They believed the red triage card substantially reduced missed cases by keeping AMI at the forefront of providers’ minds

“Basically, the card is unique, when someone sees it, it draws attention that there is something that needs to be done quickly or at a particular time.” (Nurse, M)

“…the red card draws attention and even if you don’t explain it to providers, they already know that it is associated with MI. They are all focused on getting the ECG, Troponin, et cetera.” (Nurse, M)

“The red card is a priority especially for providers working in the emergency department. That is like a visual language, before you say anything, there is already something in their minds to know that the particular patient might have MI.” (Nurse, M)

#### Provider pocket card as a clinical reminder.

The provider pocket cards were lauded by the staff as a practical reference that reinforced information they learned previously during training. Most providers were familiar with the overall approach to AMI diagnosis and treatment, but utilized the pocket card for a quick reminder of medication dosing and diagnostic thresholds. Although the pocket cards were provided both as physical laminated cards and digital copies, most providers preferred using the digital version on their phones. The pocket card was also noted to be a particularly useful educational resource for newer providers, including rotating interns.

“Pocket card is good because it is a small card with everything and makes things easier for us. It tells us what to do and what to prescribe. It helps even in reminding the new doctors that will come as well as the online training.” (Administrator, F)

“The pocket cards, they really remind us. For me I usually confuse the doses for Aspirin and Clopidogrel. There is 300 or 600, so every time I will be peeping at the card and remember. Sometimes I forget to prescribe Heparin, I will peep here again and [chuckles] then I will remember.” (Physician, F)

#### Champions facilitated evidence-based AMI care and MIMIC implementation.

Across all stakeholder groups, champions were viewed as effective in promoting adherence to MIMIC protocols and evidence-based AMI care overall. Several participants reported that champions encouraged completion of more burdensome tasks, such as the online training module, while also providing regular reminders during morning report and follow-up on individual patient cases. Participants particularly valued champions’ roles in promoting use of intervention components (such as triage cards, online training module, educational pamphlets), auditing MI care, providing regular feedback to providers, and openly recognizing high-quality clinical performance. Many providers thought the fact that the champions were already trusted colleagues and friends made them particularly effective in this role.

” They [the champions] are working well. We have different cases in the emergency department so sometimes it’s hard to remember all protocols, like doing an ECG and troponin in the first hour and again after three hours. It’s not easy if you don’t have someone who can remind you. You may find patients stay for more than three hours and are admitted with only a single ECG and troponin. So, it’s important for the champions to continue being there.” (Physician, M)

“Because the champions were supervising the implementation of all the other components. So, without champions the rest probably couldn’t be implemented well. The champions were the ones following up on placing the cards, doing an ECG, reminding that when the troponin results come out to give Aspirin, do not be afraid of giving Aspirin to patients with CKD. Reminding us to do online module. I believe the champions were very important.” (Administrator, F)

#### Online training-enhanced provider competency.

Despite concerns regarding time and internet access, participants consistently regarded the online training module as improving providers’ ability to diagnose and treat AMI. Many participants valued the module for providing straightforward direction on diagnostic test interpretation, reinforcing medication recommendations, and addressing common misconceptions, such as concerns regarding aspirin use in patients with chronic kidney disease (CKD). The online module was also noted to be beneficial for providing constructive feedback to participants.

“There are some medicines that we were so scared to prescribe but the online module assisted us in clearing those doubts.” (Nurse, F)

“It (online module) updates us for instance, you usually submit your work, so they give us feedback on what we have done. What we have achieved, what needs improvement, and the like; so, it really helps us to know where we are, and we are going. We put effort on the points that we need to improve. It is really helpful.” (Nurse, F)

“Because the online module has touched well on misconceptions, it ensures providers diagnose MI at an earlier stage. It also explains how to diagnose it from the ECG and troponin readings. It also explains what medicines to prescribe, at what dosage and at which time.” (Physician, M)

#### Inconsistent pamphlet distribution.

Participants reported that patient education pamphlets were sometimes not given to patients, which limited their overall effectiveness. Most attributed inconsistent distribution to the busy workload inherent to the ED. ED staff also commented on the pamphlet’s storage location being out of sight and therefore difficult to consistently locate and remember. Although several providers questioned whether the ED was the appropriate setting for pamphlet distribution, none cited this concern as a reason they had not given a pamphlet to a patient.

“There are two issues. You find that a person is too focused in providing care to give the pamphlet. The second thing is where they are stored. It becomes somewhat challenging to access them anytime. People don’t know where they are stored and by the time they know where the cards are the patient may have left. If it was kept in a place where everyone can see it would be easy to pick. At the emergency we have new doctors and nurses, we have new students, you understand. It becomes challenging to identify such things at that time. But if the cards are in an open space where everyone can see, When the student comes there, they will be asking what is this? They will be told that MI patients are given the pamphlet, so they know that if they get such patients, they need to give them.” (Nurse, M)

“The first hassle is in looking for them because there are many files in there. We need to keep the educational pamphlets elsewhere please. You might look and miss them sometimes.” (Physician, F)

### Self-Efficacy

#### Provider empowerment in AMI management.

Many participants stated that the MIMIC intervention effectively boosted their confidence in their ability to manage patients with AMI. This confidence stemmed from a stronger knowledge base gained through training materials such as the online module and pocket card, which helped them recognize pertinent signs and symptoms and interpret diagnostic results. After MIMIC implementation, staff noted a significant improvement in their confidence to treat AMI, particularly in administering lifesaving medications. Prior to the intervention, guidelines were often not followed; however, afterward, participants expressed a clearer understanding of the importance of each medication. They also felt comfortable prescribing these treatments and teaching others to do the same.

“I have proof, some patients I sent to [cardiac referral center] are still alive to this day. Most of my patients, all of them. I can say most of my patients they are doing good. I was able to diagnose them, give them the medication and send them for PCI and normal I make follow-up to them. I did that.” (Physician, F)

“I: After meeting the MIMIC training intervention, all the five parts, were you able to perform your part?

R: Definitely! It has been like a wake-up call for everybody that MI is a serious thing. I can perform everything to ensure that everything is there, medication is there and investigations.” (Nurse, M)

“Because one of a doctor’s values is being competent and objective. MIMIC intervention has been able to assist me with identifying these patients and channeling them to the appropriate places. It has increased something in my capacity to treat.” (Physician, M)

#### Patient self-management confidence.

Patients stated that they had more confidence in their ability to manage their heart disease after they left the hospital due to the information provided in the patient education pamphlet. They repeatedly credited the pamphlet for clearly explaining red flag symptoms and return precautions. They also emphasized that it described the medication regimen they should maintain after discharge, including when and how to take medications, as well as the timing of follow-up visits. Patients described the pamphlet as an excellent source of information and felt that it helped them manage their condition more effectively.

“Because I want to know, what did they say on the pamphlet? When I felt the chest pain-like symptoms, I said what did they say here. I liked it because I know if I experience any problem, I will go back to the pamphlet to see what it says about that problem. For instance, if I get a headache I check to see if they mentioned headache as a symptom of heart attacks.” (Patient, M)

“It has motivated me to continue using my medicines to cure this disease. If I use my medicines, it won’t be easy for that problem to recur.

[…]

It has encouraged me to go to the clinic for follow up. That is why initially I was attending the monthly clinics, but I am now only having to go after every two months. I attend the clinics and do all tests. I do ECHO, ECG, every time that I go there I do all the tests. They check my progress and prescribe me medications, so it causes me to continue receiving care, like using the medicines, and preventing another heart attack. My health will become better, and I will be doing very fine.” (Patient, M)

## Discussion

Emergency medicine is an emerging field in SSA and Tanzania specifically [18]. In recent years, there has been an increasing effort to define and track quality metrics in EDs in SSA, [19] but few projects have progressed to implementation and subsequent evaluation. To our knowledge, MIMIC is the first quality improvement intervention for AMI care in SSA [11]. Using qualitative interviews from diverse stakeholders, this study builds on previous work to provide a nuanced understanding of the factors shaping intervention acceptability and to generate actionable feedback to inform MIMIC’s expansion across Tanzania. This study complements prior quantitative evaluations of MIMIC by providing contextual insight into the mechanisms through which the intervention achieved its effects, which is essential for informed scale-up.

To evaluate the acceptability of this intervention, we used TFA, which has previously been used to evaluate acceptability of HIV-related healthcare interventions in SSA and in Tanzania specifically [20–22]. To our knowledge, this is the first application of TFA for ED-based implementation research in SSA, and we found it to be a useful framework for capturing a rich, multidimensional picture of intervention acceptability in our setting. In prior quantitative surveys using the AIM instrument, we reported that MIMIC was highly acceptable, with a mean AIM score of 4.82 out of 5 among providers and 4.68 out of 5 among patients [13,14]. However, quantitative measures alone do not capture the nuanced understanding of acceptability provided by qualitative methods. Qualitative interviews identified divergent stakeholder views on pamphlet distribution and concerns regarding internet access for the online module that were not apparent from the uniformly high AIM scores, illustrating the complementary value of qualitative methods in implementation evaluation.

The MIMIC intervention was widely viewed as acceptable and effective across all seven TFA domains. Of these, perceived effectiveness was the most frequently discussed domain of acceptability, with staff citing observable improvements in AMI recognition, diagnostic testing, and evidence-based treatment as the primary basis for their approval. These perceptions are corroborated by quantitative data from the MIMIC pilot trial, which demonstrated significant increases in ECG testing (89.4% vs. 55.3% pre-intervention), troponin testing (78.0% vs 41.4%), and AMI case identification (24.4% vs 14.9%), as well as large increases in aspirin, clopidogrel, and heparin administration among patients with confirmed AMI [12]. Importantly, these gains were sustained one year after the pilot trial concluded, with high intervention fidelity and strong normalization of MIMIC into routine clinical practice, suggesting that the improvements perceived by stakeholders reflect durable changes in care delivery [23]. Staff also described a meaningful cultural shift in the ED, with AMI now recognized as a critical emergency requiring immediate attention—a change that may be as important as the specific intervention components themselves in sustaining long-term improvements in AMI care.

The low burden perceived by participants reflects the importance of contextual tailoring in intervention design. The red triage card exemplified this principle, fitting seamlessly into the existing card-based triage system without requiring additional steps or resources. The online module was the one component that diverged from this pattern, requiring time and internet access outside working hours; however, participants still valued it highly for improving provider knowledge and correcting common misconceptions about AMI management, such as concerns about aspirin use in patients with CKD. Participants’ unanimous view that MIMIC did not detract from other clinical responsibilities is notable given the high patient volumes and resource constraints characteristic of EDs in low-income settings and suggests that pragmatic, workflow-integrated interventions can strengthen AMI care without competing with existing clinical priorities. The framing of AMI care as the highest clinical priority, rather than an additional burden, suggests that MIMIC may have contributed to a shift in clinical values at KCMC, in which evidence-based AMI management is viewed as a core emergency medicine competency rather than an optional quality improvement activity.

The high self-efficacy reported across provider stakeholder groups represents an important implementation outcome. Participants described a shift from avoidance of AMI-related diagnostics and medications due to unfamiliarity or misconceptions, to active and confident use of these tools following MIMIC implementation. This mirrors findings from provider training interventions in other low-resource settings, where knowledge gaps and misconceptions rather than resource constraints alone were identified as key drivers of suboptimal AMI care [4,5]. The pocket card and online training module appear to have been particularly effective in this regard, providing accessible, just-in-time clinical decision support that reinforced training content at the point of care. High intervention coherence, reflected in participants’ ability to clearly articulate all five MIMIC components and their intended purpose, suggests that the co-design process produced an intervention that was not only contextually appropriate but also conceptually clear to its intended users. This is consistent with broader literature demonstrating that the input of intended users in the design process promotes usability and minimizes burden [24,25]. Participants emphasized that champions reinforced these gains by providing ongoing reminders and encouraging continued use of the intervention, consistent with prior literature describing the role of clinical champions during implementation [26]. Notably, participants described feeling empowered to teach others, including rotating interns and new staff, suggesting that MIMIC may have fostered a self-sustaining learning culture within the KCMC ED that extends its impact beyond the formal intervention components.

Ethical alignment was another key driver of acceptability, as participants viewed MIMIC as consistent with their professional duty to provide high-quality, life-saving care. However, there were differing views regarding the appropriateness of distributing patient pamphlets in the ED. Providers anchored their concerns in the acuity of the clinical encounter, while patients, the intended recipients, reported no such concerns and consistently found the pamphlets empowering and informative—several describing them as filling gaps in the verbal communication they received from providers. This discordance illustrates why implementation evaluations should include patient perspectives rather than relying solely on provider perceptions when assessing patient-facing interventions. Staff concerns about the educational pamphlet likely contributed to limited fidelity to this component; in the pilot trial, only 38% of patients with AMI in the KCMC ED actually received the pamphlet [12]. Patients who did receive it described using it not only for their own self-management but also as a community education tool, sharing it with family members and social contacts to raise awareness of AMI symptoms and prevention, a ripple effect that extends the educational reach of the intervention beyond the clinical encounter. Future iterations of MIMIC should consider designating a non-physician staff member to take responsibility for pamphlet distribution, thereby removing this task from the physician’s acute care workflow, and should explore distributing pamphlets in inpatient or outpatient follow-up settings where providers have more time and patients may be more receptive. Although the patient educational pamphlet was developed through an iterative co-design process in which patients with recent AMI reviewed the patient-facing materials and provided feedback regarding comprehensibility and design, all participants in the present study were literate. Additional study is therefore needed to evaluate the acceptability of the pamphlet among patients with limited literacy or no formal education [10].

These findings suggest that intervention acceptability arose from the combination of contextual adaptation through co-design, observable improvements in clinical care, and smooth integration into existing workflows, rather than from any single intervention component alone. These findings have several implications for future implementation of MIMIC. Components identified by participants as particularly valuable, including provider training, clinical champions, and decision-support tools, require ongoing support to maintain their use over time, particularly as staff turnover introduces new clinicians without prior MIMIC training. As MIMIC expands to additional sites, continued attention to implementation fidelity and integration into routine clinical workflows will be important. Consistent with these findings, a separate evaluation of MIMIC demonstrated high perceived organizational capacity for sustainability and strong normalization of the intervention into routine clinical practice among KCMC ED clinicians, suggesting that the gains achieved during the pilot trial are likely to persist [15].

This study had several limitations. First, this study was conducted at a single tertiary referral hospital that served as both the co-design partner and implementation site for MIMIC. As such, the generalizability of these findings may be limited, as the resources, staffing, and institutional culture at KCMC may not reflect those of smaller, rural, or less-resources facilities across Tanzania. Intervention acceptability may therefore differ in these settings. Future studies should investigate the acceptability of MIMIC in diverse emergency care settings, including district and rural hospitals, to determine whether adaptation of specific intervention components is needed. Second, participant responses may have been subject to social desirability bias. This risk may have been heightened particularly given the involvement of some participants in the co-design and implementation of MIMIC, as well as by the affiliation of several investigators with the study institution, which may have influenced participants’ willingness to express criticism of the intervention. These factors could have skewed responses toward more positive expressions of acceptability. To mitigate this potential risk, interviewers underwent extensive training in best practices for in-depth interviewing to create a non-judgmental environment and encourage honest, critical feedback. Nonetheless, the acceptability of the MIMIC intervention may have been overstated, and our findings should be interpreted with this possibility in mind. Third, this study captured acceptability perceptions during the first year of implementation; views may evolve as the intervention matures or as staff turnover changes the composition of the clinical team. Longitudinal qualitative assessment would be valuable to understand how acceptability changes over time. Finally, patient participants were limited to those who had received the educational pamphlet, as only patients who received it could comment on its acceptability. No patient interviewed reported not reading the pamphlet, which stood in contrast to provider concerns that patients might not engage with it. However, because only a minority of AMI patients actually received the pamphlet, and because providers may have been less likely to distribute it to patients they perceived as less receptive, the patient perspectives elicited in this study may not be representative of the broader patient population.

In conclusion, in an ED in northern Tanzania, the MIMIC intervention was found to be acceptable across all TFA domains among providers and patients. Perceived effectiveness was the primary driver of acceptability, underpinned by observable improvements in AMI diagnosis and treatment that were corroborated by quantitative data from the pilot trial. The co-design approach used in MIMIC development likely contributed to its broad acceptability and minimal perceived burden. Future research should evaluate the acceptability, feasibility, and effectiveness of MIMIC in diverse emergency care settings in Tanzania, with particular attention to strategies for improving fidelity to patient-facing components and sustaining implementation gains over time.

## Supporting information

S1 Checklist COREQ checklist. Consolidated Criteria for Reporting Qualitative Research (COREQ) checklist for this study.
(PDF)

S1 Text Semi-structured interview guides. Semi-structured interview guides used for in-depth interviews with patients, providers, and administrators participating in the MIMIC intervention, including questions assessing intervention acceptability and feasibility.
(DOCX)

S1 File Qualitative codebook. Final codebook used to code transcripts and organize themes mapped to the Theoretical Framework of Acceptability.
(XLSX)
