# Supplementary material for: Stakeholder perceptions of the acceptability of an intervention to improve uptake of evidence-based emergency myocardial infarction care in Tanzania: A qualitative study
Source: PLoS One. 2026 Sep 25;21(9):e0357564. doi: 10.1371/journal.pone.0357564 (PMC13614563; doi:10.1371/journal.pone.0357564)
Supplement: Supplementary file 3 [file pone.0357564.s005.docx]

15 July 2026

The Editor

*PLOS ONE*

**Re: ‘Acceptability of an intervention to improve uptake of evidence-based emergency myocardial infarction care in Tanzania: A qualitative study’**

Dear Editor,

Thank you for the opportunity to revise and re-submit this manuscript for consideration as an original research article by *PLOS ONE*. We have reviewed reviewer comments and revised the manuscript carefully in response to these comments. We believe the revised manuscript is substantially improved and makes an important contribution to the limited literature on quality improvement interventions for acute myocardial infarction care in sub-Saharan Africa. Please see below a list of changes we have made to the submitted manuscript in response to this round of reviewer feedback:

1. *Reviewer #1: While the manuscript overall is well-written, there are still some minor spelling- and grammar errors in the manuscript. I believe the manuscript would benefit from another thorough reading, so that these can be changed (e.g. page 23, lines 373-374).*

We thank the reviewer for this comment. We have carefully re-read the manuscript in its entirety and corrected all identified spelling and grammar errors. The noted line has been rephrased to: “Several patients referenced inadequate doctor-patient communication throughout their hospital stay and felt that the pamphlets helped fill gaps in the information they received.”

2. *Reviewer #1: In addition, the text is phrased somewhat strongly and unnuanced at times, and I believe the text may be phrased a bit more nuanced (e.g. in the abstract, page 3, line 58, MIMIC was considered widely acceptable).*

We thank Reviewer #1 for this comment. We have reviewed the manuscript for instances of overly strong or unqualified language and revised several passages accordingly. For example, in the Abstract, we revised “MIMIC was widely acceptable in all seven TFA domains” to “MIMIC was found to be acceptable across all seven TFA domains,” and “All components were described as effective and easily integrated into existing workflows” to “Most components were described as effective and easily integrated into existing workflows.” We also softened similar language in the Results, Discussion, and Conclusion to better reflect the nuance of our findings.

3. *Reviewer #1: The use of abbreviations is mostly consistent throughout the manuscript, but not completely: consistency in the use of abbreviations would benefit the quality of the manuscript (e.g. CKD and chronic kidney disease)*

We thank Reviewer #1 for this comment. We have reviewed the manuscript for abbreviation consistency and made several revisions accordingly. For example, we defined chronic kidney disease (CKD) at first use in the body text and electrocardiogram (ECG) at first use in the Abstract. We also standardized use of “AMI” throughout the manuscript and corrected minor formatting inconsistencies, including the capitalization of NVivo 14.

4. *Reviewer #1: The opportunities to improve AMI care, in particular in the context as described throughout the manuscript, are partially related to healthcare provider’s knowledge. Nevertheless, we know that delays in seeking care for CVD/AMI are also at least partially related to patients’ symptom recognition and interpretation, which is currently overlooked. Moreover, I expect that in Tanzania, similar to other countries, access to care may be limited by physical distance. While not highly relevant to the subject at hand, I believe it is important to at least briefly mention that these factors also contribute to mortality related to AMI, before focusing on system-delays in AMI care.*

We thank Reviewer #1 for this comment. We have revised the Introduction to acknowledge that delays in AMI care are also influenced by patient symptom recognition, delays in seeking care, and barriers to healthcare access, in addition to healthcare system factors. We then focus on the provider- and system-level barriers that informed the development of the MIMIC intervention.

5. *Reviewer #1: An explanation of the Tanzanian healthcare system may help readers from an international audience to understand the context in which this study is implemented. As an international reader, I was very surprised to read that ED-staff still required training on providing AMI care in Tanzania, since this is very different from my experience in healthcare.*

We thank Reviewer #1 for this comment. We have revised the Introduction to more clearly describe prior observational and implementation research in northern Tanzania, which identified inadequate provider training, insufficient diagnostic resources, and inefficient emergency department systems as important barriers to AMI care. These findings informed the design of MIMIC and motivated the inclusion of provider education as a core intervention component.

6. *Reviewer #1: Would it be possible to provide more information on the intervention components, and/or provide the triage cards, provider pocket cards, patient education pamphlets in the appendices? I believe this may be of large value for the readers to understand what was implemented, and what these looked like (see Table 1 from* [*https://doi.org/10.1371/journal.pone.0344093*](https://doi.org/10.1371/journal.pone.0344093) *as an example).*

We thank Reviewer #1 for this helpful suggestion. We have expanded the description of the MIMIC intervention in the Introduction to provide additional detail regarding the purpose and intended function of each intervention component. The full MIMIC intervention components (including triage cards and patient educational pamphlets) have been previously published: <https://doi.org/10.5334/aogh.4361.s1>. We have added this explanation and citation to the revised Methods section.

7. *Reviewer #1: Page 5, line 88: please change AMI testing to AMI diagnostics if this is a more appropriate term.*

We thank Reviewer #1 for this suggestion. We agree that "AMI diagnostics" is a more appropriate term and have revised the text accordingly.

8. *Reviewer #1: Under setting (page 6, lines 118-121), the authors write: “At the time of the interviews, KCMC lacked a cardiologist on staff and did not have capacity for percutaneous coronary intervention or coronary surgery. AMI patients requiring such care are typically transferred to the national cardiac center in Dar es Salaam.” I believe this may be important contextual information, however, I would also like to hear the implications that this has on the study findings. Does this mean that the most severe patients are not referred to this hospital? And how would this affect the findings?*

We thank Reviewer #1 for this thoughtful comment. KCMC serves as a major referral hospital for northern Tanzania and routinely evaluates patients with suspected AMI, including those with severe disease who may ultimately require advanced cardiac care. While patients requiring PCI or cardiac surgery are subsequently referred to the national cardiac center, they are typically evaluated and initially managed at KCMC prior to transfer. Therefore, the lack of PCI and cardiac surgery capacity is unlikely to substantially affect interpretation of the present findings, as the MIMIC intervention focused on AMI recognition, diagnosis, and evidence-based medical management in the emergency department rather than advanced cardiac procedures. We have added text to clarify this point in the manuscript.

9. *Reviewer #1: Please also add the compensation in the local currency, beyond USD.*

We thank Reviewer #1 for this suggestion. We have revised the text to report participant compensation in both local currency and USD. The relevant sentence now reads: “All in-depth interview participants received 5,000 Tanzanian shillings (approximately 2 USD) as compensation for their time.”

10. *Reviewer #1: Under data collection, page 7, line 146, the authors write: “Interviews were conducted face-to-face in a private space in Swahili by Tanzanian research assistants […]”. I would rephrase this to “in Swahili, in a private space”, as this may be confusing for international readers may not know Swahili is a language.*

We thank Reviewer #1 for this suggestion. We agree that the original wording may be unclear to readers unfamiliar with Swahili and have revised the sentence accordingly. The text now reads: “Interviews were conducted face-to-face in Swahili, in a private space, by Tanzanian research assistants.”

11. *Reviewer #1: Please mention what quality assurance measures were taken, and if none were taken, why this was not done.*

We thank Reviewer #1 for this comment. We have added additional detail to the Methods describing the procedures used throughout the coding and analysis process, including regular study team meetings to review coding decisions, refine the codebook, and ensure consistent application of codes across transcripts.

12. *Reviewer #1: Under data analysis, page 9, line 188, the authors write: “Dominant themes were identified by respondent group and by the number of respondents reporting each theme”. I understand that different themes may have been dominant/relevant for different participant subgroups, but the number of respondents/participants reporting a theme appears to be quantifying the findings, which is not necessary for this type of work.*

We thank Reviewer #1 for this thoughtful comment. We agree that the original wording may have implied that themes were identified solely on the basis of participant counts. While the prevalence of themes across interviews informed our assessment of thematic prominence, dominant themes were identified through iterative review of coded transcripts and consideration of their relevance to intervention acceptability. We have revised the text to clarify this point.

13. *Reviewer #1: Related, please be consistent in the use of respondent or participant.*

We thank Reviewer #1 for this suggestion. We agree and have revised the manuscript for consistency, replacing “respondent” with “participant” throughout.

14. *Reviewer #1: Under ethical considerations, (page 9, line 197), I am missing some information. Typically, I would expect to read what happens to the audio files, what happens to the transcripts, what happens to the patient information, and how this is all done securely, where and how long data is stored for, etc.*

We thank the reviewer for this suggestion. We have expanded the Ethical Considerations section to provide additional information regarding management of the qualitative data. Specifically, we now clarify that interviews were audio-recorded with participant consent, transcripts were de-identified prior to analysis, and study data were stored securely with access limited to authorized study personnel. We also note that data were managed in accordance with institutional and ethics committee requirements. We also specifically mention that study data will be stored on a secure server at KCMC for 10 years, as per Tanzania National Institute for Medical Research requirements.

15. *Reviewer #1: If the interviewers/researchers had previous contacts with the healthcare providers (or patients) who were interviewed, I would appreciate this being mentioned briefly in the methods for the sake of transparency.*

We thank the reviewer for this helpful suggestion. We have added additional detail to the Methods section describing the relationship between the research team and study participants. Specifically, we clarify that some members of the research team had prior professional relationships with KCMC ED staff participants, while interviews were conducted by Tanzanian research assistants with no prior supervisory, evaluative, or clinical relationship with any participant.

16. *Reviewer #1: The themes are presented clear with supporting quotes in-text. I was wondering whether there were only themes, and if no sub-themes emerged during the process. I am typically used to a larger code-three and wonder if the data does not contain more in-depth information which is currently not provided as subthemes.*

We thank Reviewer #1 for this observation. Our analysis was structured deductively around the seven domains of the TFA, which serve as the superordinate organizing framework, with 19 discrete themes identified within those domains. The TFA domains and themes therefore constitute a two-level hierarchy. Subthemes were not identified as a separate level in our analysis, as the 19 themes adequately captured the depth of the data within each TFA domain. We have added a clarifying sentence to the Data Analysis section to make this structure more explicit.

17. *Reviewer #1: Page 29, line 522: the word transplant may be better replaced by ‘implement’ or another adjective.*

We thank the reviewer for this comment. We have updated the manuscript wording accordingly.

18. *Reviewer #1: As becomes clear in the results and discussion, some participants from this study were involved in the development of the intervention, and some of the authors work at the same institution as where the intervention was implemented, which may further lead to social desirability bias. Both are mentioned very briefly as a limitation, but I believe this is a large limitation that should be addressed more thoroughly, in particular how this may effected the findings, and what the implications of this may be.*

We thank Reviewer #1 for this insightful comment. We have expanded the Limitations section to further discuss the potential for social desirability bias. Specifically, we discuss how participant involvement in the co-design process and investigator affiliations with the study institution may have contributed to more favorable perceptions of the intervention and the implications of this potential bias for interpretation of the findings.

19. *Reviewer #1: On page 43, line 822, the authors write: “Additional study is needed to explore acceptability of the pamphlets in a broader patient population, including among patients who have limited literacy.” While I completely agree this is highly relevant, this is the first time literacy is mentioned. Do we know anything about the literacy or educational levels of the patients involved? Were the pamphlets not co-created with a group of individuals with varying levels of health literacy, since I would expect the authors to have done so.*

We thank the reviewer for this helpful comment. As the reviewer notes, the patient educational pamphlet was developed through an iterative co-design process in which five patients recently admitted with AMI reviewed the educational materials and provided feedback regarding their comprehensibility and design. However, all 5 patient participants in the present study were literate. We have now clarified the literacy of our participants in the revised Results section. We have also revised the Discussion to clarify the co-design process while noting that additional study is needed to evaluate the acceptability of the pamphlet among patients with limited literacy, particularly those with no formal education.

20. *Reviewer #1: I would remove this sentence: “Nonetheless, the consistency and specificity of responses support the credibility of the findings”, since this does not mitigate the limitations.*

We thank the reviewer for this comment. We have removed the aforementioned sentence from the Limitations section.

21. *Reviewer #1: I would appreciate a more critical discussion on the generalizability of the findings from this setting to other settings, including smaller or more rural hospitals, hospitals with more limited means, and otherwise different circumstances. Since this intervention was both co-created with individuals from this hospital, as well as implemented in this hospital, the generalizability may be a large limitation of the current study, but this is only briefly mentioned.*

We thank Reviewer #1 for this helpful comment. We have expanded the Limitations section to further discuss the generalizability of these findings. Specifically, we note that MIMIC was co-designed and evaluated at a single tertiary referral hospital with an established emergency medicine program and that the generalizability of these findings may be limited, as intervention acceptability may differ in smaller, rural, or less-resourced facilities across Tanzania. We also discuss the need for future evaluation in diverse emergency care settings to determine whether adaptation of specific intervention components is needed.

22. *Reviewer #1: Is there any quantitative data on improvements in effectiveness and efficiency? If so, I would appreciate this information in the discussion.*

We thank the reviewer for this helpful suggestion. We have revised the Discussion to include a brief summary of previously published quantitative evaluations of the MIMIC intervention, including improvements in effectiveness, implementation outcomes, and long-term sustainability. Specifically, we cite previously published studies describing effectiveness on clinical performance metrics (Hertz et al, *BMJ Open*, 2025), long-term implementation and effectiveness (Wang et al., *Ann Glob Health.* 2026), implementation outcomes including fidelity and penetration (Hertz et al., *Ann Glob Health.* 2025), and sustainability and normalization (Wang et al., *Implement Sci Commun.* 2026).

23. *Reviewer #1: I would also appreciate a critical reflection on the sustainability of MIMIC: what are the costs, is it possible and feasible to have long-term champions? What if the current Champions stop or leave? What are barriers to long-term implementation and sustainability in the current setting, and in other settings?*

We thank Reviewer #1 for this thoughtful comment. While sustainability was not a primary focus of the present study, we agree that it is an important consideration. We have added discussion linking the present findings to our recently published study demonstrating high perceived organizational capacity for sustainability and strong normalization of MIMIC among KCMC ED clinicians (Wang et al., *Implement Sci Commun.* 2026).

24. *Reviewer #2: The title has been evaluated for clarity and length. However, it does not align with the study's aim and methodological approach. Since this is a qualitative study that explores perceptions and experiences, as indicated in lines 100-102, it is recommended that the title more accurately reflect both the approach and the aim of the research.*

We thank Reviewer #2 for this feedback. We agree that the title should reflect both the qualitative nature of the study as well as its focus on stakeholder perceptions of intervention acceptability. As such, we have adjusted the title to the following: “Stakeholder perceptions of the acceptability of an intervention to improve uptake of evidence-based emergency myocardial infarction care in Tanzania: A qualitative study.”

25. *Reviewer #2: Authors information was clear.*

We thank the reviewer for this positive comment.

26. *Reviewer #2: Line 35 The abstract is clear and meets the journal's 298-word limit (300). However, adding subheadings such as 'Background', 'Purpose', and so on would improve clarity.*

We thank the reviewer for this comment. We have added subheadings to the abstract to improve clarity.

27. *Reviewer #2: The purpose of the study was not clearly written in the abstract.*

We thank Reviewer #2 for this astute observation. Accordingly, we have clearly elaborated on the study aim in the abstract with the following sentence: “the aim of this study was to explore stakeholder perceptions of the acceptability of MIMIC following its implementation.”

28. *Reviewer #2: Line 38 until 40 “was developed and implemented in the emergency department (ED) of a regional referral center in northern Tanzania.” Better to place it under the method part as it is relevant to the setting of the study.*

We thank the reviewer for this feedback. We agree that a description of the study setting is better suited to the Methods section of the abstract; we have revised the abstract accordingly.

29. *Reviewer #2: Line 42 it is better to write the approach of the study “A qualitative study conducted...”*

We thank Reviewer #2 for this suggestion. We have revised the opening sentence of the Methods section to highlight the qualitative study design.

30. *Reviewer #2: Line 43 did you mean you have included a sample of patients diagnosed with MI, kindly clarify it.*

We thank the reviewer for this question. We have clarified that key stakeholders included patients diagnosed with AMI who participated in MIMIC during its first year of implementation.

31. *Reviewer #2: Overall, introduction was written clearly describing the information needed. Kindly, make sure to use Vancouver styles in writing the references following journal guidelines.*

We thank the reviewer for this comment. We have reviewed the manuscript and revised the references and in-text citations to ensure they are consistent with Vancouver style and the journal's formatting guidelines.

32. *Reviewer #2: Line 100 the aim of the study is not consistent with the title and the aim of the study, using different objective may confuse the readers, be specific and consistent (acceptability, feasibility, perception)*

We thank Reviewer #2 for this comment. We agree that consistency between the title, study objectives, and analytic framework is important. We have revised the study aim to clarify that the primary objective was to examine the acceptability of the MIMIC intervention and to better understand factors shaping intervention acceptability among key stakeholder groups.

33. *Reviewer #2: The method section is clear and sufficient. However, the authors did not explicitly address trustworthiness—credibility, transferability, dependability, and confirmability—which is essential in qualitative studies. State directly, for example, that credibility was established through prolonged engagement during interviews (line 149: Each interview lasted approximately 45-60 minutes). Also, clarify that triangulation was used, such as multiple data sources, investigators, or methods to cross-check findings (line 140: developed by the international, interdisciplinary investigator team).*

We thank Reviewer #2 for this feedback. We agree that the manuscript should more explicitly	describe strategies used to enhance trustworthiness. We have revised the Methods section to	address credibility, transferability, dependability, and confirmability, including purposive	sampling across multiple stakeholder groups, independent coding by a diverse research team,	iterative codebook refinement, and consensus-based resolution of discrepancies.

34. *Reviewer #2: Line 167 is an excellent example of applying the trustworthiness or rigorousness. specifically, it contributes to dependability and credibility.*

We thank the reviewer for highlighting this example.

35. *Reviewer #2: Line 109 it is better to keep the setting name anonymous to ensure privacy.*

We thank the reviewer for this suggestion. We have respectfully elected to retain the study site	identifier, as we believe the institutional context is crucial for interpreting the implementation	setting and assessing the transferability of our findings. We have ensured participant	confidentiality by omitting all participant-identifying information from the manuscript. We	believe that naming the study site provides important context without compromising participant	confidentiality.

36. *Reviewer #2: Line 126 kindly give more details about the eligibility criteria to ensure the purposive sampling method such as Specific roles or characteristics (e.g., job title, diagnosis, age minimum)*

We thank Reviewer #2 for this helpful suggestion. We have expanded the Methods section to provide additional detail regarding the eligibility criteria and purposive sampling strategy. Specifically, we now describe the inclusion criteria for each stakeholder group, including provider roles and patient eligibility (age ≥18 years and confirmed AMI diagnosis), and clarify how participants were purposively selected from each stakeholder group.

37. *Reviewer #2: Line 127 did you mean you have included patients with AMI who were in ED during the study. How long usually patients stay there.*

We thank Reviewer #2 for this comment. To clarify, patients were not interviewed while	receiving AMI care in the emergency department. Eligible patients were patients diagnosed with	AMI in the ED during the study period, who were subsequently contacted by telephone after	hospital discharge and invited to participate in an in-depth interview. We have revised the	Methods section to clarify the eligibility and recruitment process.

38. *Reviewer #2: Analysis was written thoroughly and well.*

We thank the reviewer for this positive feedback.

39. *Reviewer #2: Line 222 the table of the themes well written, clear, and included detailed information.*

We thank the reviewer for this encouraging remark.

40. *Reviewer #2: Q: why in some themes patients did not involve. Specifically in some themes related burden, cost and effectiveness of the intervention, justify it kindly.*

We thank the reviewer for this comment. Patients only interacted directly with the patient educational pamphlet, whereas the remaining components of the intervention were directed toward healthcare providers. As a result, patients were unable to comment on certain themes related to provider-facing components of the intervention. We have clarified this point in the manuscript.

41. *Reviewer #2: Note: Several themes are overly lengthy. Shorter phrases are recommended to enhance readability, maintain focus, and facilitate comparison. For instance, the theme 'Concerns about internet access and time needed to complete the online training module' could be condensed to 'Technological and time barriers,' 'Access and time concerns,' or 'Time and connectivity challenges.' It is advisable to shorten the descriptive themes accordingly.*

We thank Reviewer #2 for this helpful suggestion. We agree that more concise theme names	enhance readability and facilitate comparison across the Theoretical Framework of Acceptability	domains. Accordingly, we have revised all of the theme titles throughout Table 2 and the	corresponding Results section for concision while preserving their original intended meaning.

42. *Reviewer #2: Note: many of the themes related to the effectiveness can merged due to similarities. It is suggested to consolidate similar themes related to effectiveness to minimize repetition and maintain conciseness.*

We thank the reviewer for this feedback. We carefully reviewed the themes for redundancy and determined that, although several themes address related aspects of intervention effectiveness, they represent conceptually distinct findings with different participant frequencies and stakeholder distributions. We therefore retained the original thematic structure while revising the theme titles for improved clarity and concision. In addition, to reduce repetition, maintain concision, and improve readability, we heavily revised several sub-sections of the Results to streamline the presentation of related findings.

43. *Reviewer #2: Line 243 “…...” (Physician, M)” kindly use labeling participant quotes with an identifier for all participants. it is standard practice and highly recommended to label each participant quote with a unique identifier in qualitative study to ensure transparency and credibility and Allows readers to see the range of views across your sample.*

We thank the reviewer for this suggestion. We have respectfully elected to retain our current	approach to labeling participant quotations by stakeholder group (i.e. physician, administrator,	etc) and gender, as we believe it provides sufficient contextual information while upholding	patient confidentiality given the small sample size within each stakeholder group.

44. *Reviewer #2: Line 270 use the conation marks “I really… routine.”*

We thank the reviewer for this helpful feedback. We have added the missing quotation marks and reviewed the remainder of the manuscript to ensure that all participant quotations are appropriately formatted.

45. *Reviewer #2: Discussion was not written thoroughly and was not covered all themes. Authors do not need to list the results again but interpret and connect them to previous literature, compare and contrast.*

We thank the reviewer for this constructive feedback. We have substantially revised the Discussion in response to these comments. Specifically, we reorganized the Discussion to focus more on interpretation of our findings in the context of prior literature rather than restating the Results. We added new discussion of previously underdeveloped themes, including self-efficacy, opportunity costs, intervention coherence, and burden, and expanded our interpretation of these findings by relating them to prior literature on co-design, clinical champions, provider training, and health literacy interventions. Finally, we added discussion of the complementary role of the Theoretical Framework of Acceptability relative to quantitative measures of acceptability. We believe these revisions strengthen the interpretation of our findings and better contextualize them within the existing literature.

Thank you for your consideration.

Sincerely,
Janet Bettger, ScD, MS, FAHA

Professor and Vice Chair of Research
Department of Physical Medicine and Rehabilitation, UNC School of Medicine
