# Supplementary material for: Stakeholder perceptions of the acceptability of an intervention to improve uptake of evidence-based emergency myocardial infarction care in Tanzania: A qualitative study
Source: PLoS One. 2026 Sep 25;21(9):e0357564. doi: 10.1371/journal.pone.0357564 (PMC13614563; doi:10.1371/journal.pone.0357564)
Supplement: Peer review history

# Peer review: Decision Letter 0

**DOI:** 10.1371/journal.pone.0357564.r001

**Authors:** Buna Bhandari

9 Jun 2026

Dear Dr. Bettger,

Thank you for submitting your manuscript to PLOS ONE. After careful consideration, we feel that it has merit but does not fully meet PLOS ONE’s publication criteria as it currently stands. Therefore, we invite you to submit a revised version of the manuscript that addresses the points raised during the review process.

Please submit your revised manuscript by Jul 24 2026 11:59PM. If you will need more time than this to complete your revisions, please reply to this message or contact the journal office at plosone@plos.org. When you're ready to submit your revision, log on to https://www.editorialmanager.com/pone/ and select the 'Submissions Needing Revision' folder to locate your manuscript file.

- A letter that responds to each point raised by the academic editor and reviewer(s). You should upload this letter as a separate file labeled 'Response to Reviewers'.
- A marked-up copy of your manuscript that highlights changes made to the original version. You should upload this as a separate file labeled 'Revised Manuscript with Track Changes'.
- An unmarked version of your revised paper without tracked changes. You should upload this as a separate file labeled 'Manuscript'.

If you would like to make changes to your financial disclosure, please include your updated statement in your cover letter. Guidelines for resubmitting your figure files are available below the reviewer comments at the end of this letter.

If applicable, we recommend that you deposit your laboratory protocols in protocols.io to enhance the reproducibility of your results. Protocols.io assigns your protocol its own identifier (DOI) so that it can be cited independently in the future. For instructions see: https://journals.plos.org/plosone/s/submission-guidelines#loc-laboratory-protocols. Additionally, PLOS ONE offers an option for publishing peer-reviewed Lab Protocol articles, which describe protocols hosted on protocols.io. Read more information on sharing protocols at https://plos.org/protocols?utm_medium=editorial-email&utm_source=authorletters&utm_campaign=protocols.

As the corresponding author, your ORCID iD is verified in the submission system and will appear in the published article. PLOS supports the use of ORCID, and we encourage all coauthors to register for an ORCID iD and use it as well. Please encourage your coauthors to verify their ORCID iD within the submission system before final acceptance, as unverified ORCID iDs will not appear in the published article. *Only*  the individual author can complete the verification step; PLOS staff *cannot*  verify ORCID iDs on behalf of authors.

We look forward to receiving your revised manuscript.

Kind regards,

Dr Buna Bhandari

Academic Editor

PLOS One

Journal requirements:

When submitting your revision, we need you to address these additional requirements.

1. Please ensure that your manuscript meets PLOS ONE's style requirements, including those for file naming. The PLOS ONE style templates can be found at

https://journals.plos.org/plosone/s/file?id=wjVg/PLOSOne_formatting_sample_main_body.pdf and

https://journals.plos.org/plosone/s/file?id=ba62/PLOSOne_formatting_sample_title_authors_affiliations.pdf

2. Please include a complete copy of PLOS’ questionnaire on inclusivity in global research in your revised manuscript. Our policy for research in this area aims to improve transparency in the reporting of research performed outside of researchers’ own country or community. The policy applies to researchers who have travelled to a different country to conduct research, research with Indigenous populations or their lands, and research on cultural artefacts. The questionnaire can also be requested at the journal’s discretion for any other submissions, even if these conditions are not met.  Please find more information on the policy and a link to download a blank copy of the questionnaire here: https://journals.plos.org/plosone/s/best-practices-in-research-reporting. Please upload a completed version of your questionnaire as Supporting Information when you resubmit your manuscript.

3. In the online submission form you indicate that your data is not available for proprietary reasons and have provided a contact point for accessing this data. Please note that your current contact point is a co-author on this manuscript. According to our Data Policy, the contact point must not be an author on the manuscript and must be an institutional contact, ideally not an individual. Please revise your data statement to a non-author institutional point of contact, such as a data access or ethics committee, and send this to us via return email. Please also include contact information for the third party organization, and please include the full citation of where the data can be found.

If the reviewer comments include a recommendation to cite specific previously published works, please review and evaluate these publications to determine whether they are relevant and should be cited. There is no requirement to cite these works unless the editor has indicated otherwise.

[Note: HTML markup is below. Please do not edit.]

Reviewers' comments:

Reviewer's Responses to Questions

**Comments to the Author**

1. Is the manuscript technically sound, and do the data support the conclusions?

Reviewer #1: Yes

Reviewer #2: Yes

**********

2. Has the statistical analysis been performed appropriately and rigorously?

Reviewer #1: N/A

Reviewer #2: N/A

**********

3. Have the authors made all data underlying the findings in their manuscript fully available?

The PLOS Data policy

Reviewer #1: No

Reviewer #2: Yes

**********

4. Is the manuscript presented in an intelligible fashion and written in standard English?

Reviewer #1: Yes

Reviewer #2: Yes

**********

Reviewer #1: Dear editor,

Thank you for inviting me to review the manuscript entitled “Acceptability of an intervention to improve uptake of evidence-based emergency myocardial infarction care in Tanzania: A qualitative study Acceptability of AMI care intervention in Tanzania”. I believe this is a highly relevant and interesting article, and agree with the reviewers that a qualitative evaluation is important to provide additional insights beyond the quantitative evaluation. I believe this study is eligible for publication in PLOS One after some major revisions.

Language & wording

- While the manuscript overall is well-written, there are still some minor spelling- and grammar errors in the manuscript. I believe the manuscript would benefit from another thorough reading, so that these can be changed (e.g. page 23, lines 373-374).

- In addition, the text is phrased somewhat strongly and unnuanced at times, and I believe the text may be phrased a bit more nuanced (e.g. in the abstract, page 3, line 58, MIMIC was considered widely acceptable).

- The use of abbreviations is mostly consistent throughout the manuscript, but not completely: consistency in the use of abbreviations would benefit the quality of the manuscript (e.g. CKD and chronic kidney disease)

Introduction

- The opportunities to improve AMI care, in particular in the context as described throughout the manuscript, are partially related to healthcare provider’s knowledge. Nevertheless, we know that delays in seeking care for CVD/AMI are also at least partially related to patients’ symptom recognition and interpretation, which is currently overlooked. Moreover, I expect that in Tanzania, similar to othe countries, access to care may be limited by physical distance. While not highly relevant to the subject at hand, I believe it is important to at least briely mention that these factors also contribute to mortality related to AMI, before focusing on system-delays in AMI care.

- An explanation of the Tanzanian healthcare system may help readers from an international audience to understand the context in which this study is implemented. As an international reader, I was very surprised to read that ED-staff still required training on providing AMI care in Tanzania, since this is very different from my experience in healthcare.

- Would it be possible to provide more information on the intervention components, and/or provide the triage cards, provider pocket cards, patient education pamphlets in the appendices? I believe this may be of large value for the readers to understand what was implemented, and what these looked like (see Table 1 from https://doi.org/10.1371/journal.pone.0344093 as an example).

- Page 5, line 88: please change AMI testing to AMI diagnostics if this is a more appropriate term.

Methods

- Under setting (page 6, lines 118-121), the authors write: “At the time of the interviews, KCMC lacked a cardiologist on staff and did not have capacity for percutaneous coronary intervention or coronary surgery. AMI patients requiring such care are typically transferred to the national cardiac center in Dar es Salaam.” I believe this may be important contextual information, however, I would also like to hear the implications that this has on the study findings. Does this mean that the most severe patients are not referred to this hospital? And how would this affect the findings?

- Please also add the compensation in the local currency, beyond USD.

- Under data collection, page 7, line 146, the authors write: “Interviews were conducted face-to-face in a private space in Swahili by Tanzanian research assistants […]”. I would rephrase this to “in Swahili, in a private space”, as this may be confusing for international readers may not know Swahili is a language.

- Please mention what quality assurance measures were taken, and if none were taken, why this was not done.

- Under data analysis, page 9, line 188, the authors write: “Dominant themes were identified by respondent group and by the number of respondents reporting each theme”. I understand that different themes may have been dominant/relevant for different participant subgroups, but the number of respondents/participants reporting a theme appears to be quantifying the findings, which is not necessary for this type of work.

- Related, please be consistent in the use of respondent or participant.

- Under ethical considerations, (page 9, line 197), I am missing some information. Typically, I would expect to read what happens to the audio files, what happens to the transcripts, what happens to the patient information, and how this is all done securely, where and how long data is stored for, etc.

- If the interviewers/researchers had previous contacts with the healthcare providers (or patients) who were interviewed, I would appreciate this being mentioned briefly in the methods for the sake of transparency.

Results

- The themes are presented clear with supporting quotes in-text. I was wondering whether there were only themes, and if no sub-themes emerged during the process. I am typically used to a larger code-three, and wonder if the data does not contain more in-depth information which is currently not provided as subthemes.

- Page 29, line 522: the word transplant may be better replaced by ‘implement’ or another adjective.

Discussion

- As becomes clear in the results and discussion, some participants from this study were involved in the development of the intervention, and some of the authors work at the same institution as where the intervention was implemented, which may further lead to social desirability bias. Both are mentioned very briefly as a limitation, but I believe this is a large limitation that should be addressed more thoroughly, in particular how this may affected the findings, and what the implications of this may be.

- On page 43, line 822, the authors write: “Additional study is needed to explore acceptability of the pamphlets in a broader patient population, including among patients who have limited literacy.”. While I completely agree this is highly relevant, this is the first time literacy is mentioned. Do we know anything about the literacy or educational levels of the patients involved? Were the pamphlets not co-created with a group of individuals with varying levels of health literacy, since I would expect the authors to have done so.

- I would remove this sentence: “Nonetheless, the consistency and specificity of responses support the credibility of the findings”, since this does not mitigate the limitations.

- I would appreciate a more critical discussion on the generalizability of the findings from this setting to other settings, including smaller or more rural hospitals, hospitals with more limited means, and otherwise different circumstances. Since this intervention was both co-created with individuals from this hospital, as well as implemented in this hospital, the generalizability may be a large limitation of the current study, but this is only briefly mentioned.

- Is there any quantitative data on improvements in effectiveness and efficiency? If so, I would appreciate this information in the discussion.

- I would also appreciate a critical reflection on the sustainability of MIMIC: what are the costs, is it possible and feasible to have long-term champions? What if the current Champions stop or leave? What are barriers to long-term implementation and sustainability in the current setting, and in other settings?

Reviewer #2: Review comments of the Acceptability of an intervention to improve uptake of evidence-based emergency myocardial infarction care in Tanzania: A qualitative study.

I’m deeply grateful to all authors for their dedication, expertise, and hard work throughout this study, and hope this work inspires continued innovation and collaboration in implementation science.

The title has been evaluated for clarity and length. However, it does not align with the study's aim and methodological approach. Since this is a qualitative study that explores perceptions and experiences, as indicated in lines 100-102, it is recommended that the title more accurately reflect both the approach and the aim of the research.

Authors information was clear.

Line 35 The abstract is clear and meets the journal's 298-word limit (300). However, adding subheadings such as 'Background', 'Purpose', and so on would improve clarity.

The purpose of the study was not clearly written in the abstract.

Line 38 until 40 “was developed and implemented in the emergency department (ED) of a regional referral center in northern Tanzania.” Better to place it under the method part as it is relevant to the setting of the study.

Line 42 it is better to write the approach of the study “A qualitative study conducted...”

Line 43 did you mean you have included a sample of patients diagnosed with MI, kindly clarify it.

Introduction

Overall, introduction was written clearly describing the information needed. Kindly, make sure to use Vancouver styles in writing the references following journal guidelines.

Line 100 the aim pf the study is not consistent with the title and the aim of the study, using different objective may confuse the readers, be specific and consistent (acceptability, feasibility, perception)

Materials And Method

The method section is clear and sufficient. However, the authors did not explicitly address trustworthiness—credibility, transferability, dependability, and confirmability—which is essential in qualitative studies. State directly, for example, that credibility was established through prolonged engagement during interviews (line 149: Each interview lasted approximately 45-60 minutes). Also, clarify that triangulation was used, such as multiple data sources, investigators, or methods to cross-check findings (line 140: developed by the international, interdisciplinary investigator team).

Line 167 is an excellent example of applying the trustworthiness or rigorousness. specifically, it contributes to dependability and credibility.

Line 109 it is better to keep the setting name anonymous to ensure privacy.

Line 126 kindly give more details about the eligibility criteria to ensure the purposive sampling method such as Specific roles or characteristics (e.g., job title, diagnosis, age minimum)

Line 127 did you mean you have included patients with AMI who were in ED during the study. How long usually patients stay there.

Study Analysis

Analysis was written thoroughly and well.

Study Results

Line 222 the table of the themes well written, clear, and included detailed information.

Q: why in some themes patients did not involve. Specifically in some themes related burden, cost and effectiveness of the intervention, justify it kindly.

Note: Several themes are overly lengthy. Shorter phrases are recommended to enhance readability, maintain focus, and facilitate comparison. For instance, the theme 'Concerns about internet access and time needed to complete the online training module' could be condensed to 'Technological and time barriers,' 'Access and time concerns,' or 'Time and connectivity challenges.' It is advisable to shorten the descriptive themes accordingly.

Note: many of the themes related to the effectiveness can merged due to similarities. It is suggested to consolidate similar themes related to effectiveness to minimize repetition and maintain conciseness.

Line 243 “…...” (Physician, M)” kindly use labeling participant quotes with an identifier for all participants. it is standard practice and highly recommended to label each participant quote with a unique identifier in qualitative study to ensure transparency and credibility and Allows readers to see the range of views across your sample.

Line 270 use the conation marks “I really… routine.”

Discussion

Discussion was not written thoroughly and was not covered all themes. Authors do not need to list the results again but interpret and connect them to previous literature, compare and contrast.

**********

what does this mean?). If published, this will include your full peer review and any attached files.

If you choose “no”, your identity will remain anonymous but your review may still be made public.

**Do you want your identity to be public for this peer review?**  For information about this choice, including consent withdrawal, please see our Privacy Policy

Reviewer #1: No

Reviewer #2: No

**********

[NOTE: If reviewer comments were submitted as an attachment file, they will be attached to this email and accessible via the submission site. Please log into your account, locate the manuscript record, and check for the action link "View Attachments". If this link does not appear, there are no attachment files.]

To ensure your figures meet our technical requirements, please review our figure guidelines: https://journals.plos.org/plosone/s/figures

You may also use PLOS’s free figure tool, NAAS, to help you prepare publication quality figures: https://journals.plos.org/plosone/s/figures#loc-tools-for-figure-preparation.

NAAS will assess whether your figures meet our technical requirements by comparing each figure against our figure specifications.

# Author comment: Author response to Decision Letter 1

**DOI:** 10.1371/journal.pone.0357564.r002

9 Aug 2026

Thank you for the opportunity to revise and re-submit this manuscript for consideration as an original research article by PLOS ONE. We have reviewed reviewer comments and revised the manuscript carefully in response to these comments. We believe the revised manuscript is substantially improved and makes an important contribution to the limited literature on quality improvement interventions for acute myocardial infarction care in sub-Saharan Africa. Please see below a list of changes we have made to the submitted manuscript in response to this round of reviewer feedback:

1. Reviewer #1: While the manuscript overall is well-written, there are still some minor spelling- and grammar errors in the manuscript. I believe the manuscript would benefit from another thorough reading, so that these can be changed (e.g. page 23, lines 373-374).

We thank the reviewer for this comment. We have carefully re-read the manuscript in its entirety and corrected all identified spelling and grammar errors. The noted line has been rephrased to: “Several patients referenced inadequate doctor-patient communication throughout their hospital stay and felt that the pamphlets helped fill gaps in the information they received.”

2. Reviewer #1: In addition, the text is phrased somewhat strongly and unnuanced at times, and I believe the text may be phrased a bit more nuanced (e.g. in the abstract, page 3, line 58, MIMIC was considered widely acceptable).

We thank Reviewer #1 for this comment. We have reviewed the manuscript for instances of overly strong or unqualified language and revised several passages accordingly. For example, in the Abstract, we revised “MIMIC was widely acceptable in all seven TFA domains” to “MIMIC was found to be acceptable across all seven TFA domains,” and “All components were described as effective and easily integrated into existing workflows” to “Most components were described as effective and easily integrated into existing workflows.” We also softened similar language in the Results, Discussion, and Conclusion to better reflect the nuance of our findings.

3. Reviewer #1: The use of abbreviations is mostly consistent throughout the manuscript, but not completely: consistency in the use of abbreviations would benefit the quality of the manuscript (e.g. CKD and chronic kidney disease)

We thank Reviewer #1 for this comment. We have reviewed the manuscript for abbreviation consistency and made several revisions accordingly. For example, we defined chronic kidney disease (CKD) at first use in the body text and electrocardiogram (ECG) at first use in the Abstract. We also standardized use of “AMI” throughout the manuscript and corrected minor formatting inconsistencies, including the capitalization of NVivo 14.

4. Reviewer #1: The opportunities to improve AMI care, in particular in the context as described throughout the manuscript, are partially related to healthcare provider’s knowledge. Nevertheless, we know that delays in seeking care for CVD/AMI are also at least partially related to patients’ symptom recognition and interpretation, which is currently overlooked. Moreover, I expect that in Tanzania, similar to other countries, access to care may be limited by physical distance. While not highly relevant to the subject at hand, I believe it is important to at least briefly mention that these factors also contribute to mortality related to AMI, before focusing on system-delays in AMI care.

We thank Reviewer #1 for this comment. We have revised the Introduction to acknowledge that delays in AMI care are also influenced by patient symptom recognition, delays in seeking care, and barriers to healthcare access, in addition to healthcare system factors. We then focus on the provider- and system-level barriers that informed the development of the MIMIC intervention.

5. Reviewer #1: An explanation of the Tanzanian healthcare system may help readers from an international audience to understand the context in which this study is implemented. As an international reader, I was very surprised to read that ED-staff still required training on providing AMI care in Tanzania, since this is very different from my experience in healthcare.

We thank Reviewer #1 for this comment. We have revised the Introduction to more clearly describe prior observational and implementation research in northern Tanzania, which identified inadequate provider training, insufficient diagnostic resources, and inefficient emergency department systems as important barriers to AMI care. These findings informed the design of MIMIC and motivated the inclusion of provider education as a core intervention component.

6. Reviewer #1: Would it be possible to provide more information on the intervention components, and/or provide the triage cards, provider pocket cards, patient education pamphlets in the appendices? I believe this may be of large value for the readers to understand what was implemented, and what these looked like (see Table 1 from https://doi.org/10.1371/journal.pone.0344093 as an example).

We thank Reviewer #1 for this helpful suggestion. We have expanded the description of the MIMIC intervention in the Introduction to provide additional detail regarding the purpose and intended function of each intervention component. The full MIMIC intervention components (including triage cards and patient educational pamphlets) have been previously published: https://doi.org/10.5334/aogh.4361.s1. We have added this explanation and citation to the revised Methods section.

7. Reviewer #1: Page 5, line 88: please change AMI testing to AMI diagnostics if this is a more appropriate term.

We thank Reviewer #1 for this suggestion. We agree that "AMI diagnostics" is a more appropriate term and have revised the text accordingly.

8. Reviewer #1: Under setting (page 6, lines 118-121), the authors write: “At the time of the interviews, KCMC lacked a cardiologist on staff and did not have capacity for percutaneous coronary intervention or coronary surgery. AMI patients requiring such care are typically transferred to the national cardiac center in Dar es Salaam.” I believe this may be important contextual information, however, I would also like to hear the implications that this has on the study findings. Does this mean that the most severe patients are not referred to this hospital? And how would this affect the findings?

We thank Reviewer #1 for this thoughtful comment. KCMC serves as a major referral hospital for northern Tanzania and routinely evaluates patients with suspected AMI, including those with severe disease who may ultimately require advanced cardiac care. While patients requiring PCI or cardiac surgery are subsequently referred to the national cardiac center, they are typically evaluated and initially managed at KCMC prior to transfer. Therefore, the lack of PCI and cardiac surgery capacity is unlikely to substantially affect interpretation of the present findings, as the MIMIC intervention focused on AMI recognition, diagnosis, and evidence-based medical management in the emergency department rather than advanced cardiac procedures. We have added text to clarify this point in the manuscript.

9. Reviewer #1: Please also add the compensation in the local currency, beyond USD.

We thank Reviewer #1 for this suggestion. We have revised the text to report participant compensation in both local currency and USD. The relevant sentence now reads: “All in-depth interview participants received 5,000 Tanzanian shillings (approximately 2 USD) as compensation for their time.”

10. Reviewer #1: Under data collection, page 7, line 146, the authors write: “Interviews were conducted face-to-face in a private space in Swahili by Tanzanian research assistants […]”. I would rephrase this to “in Swahili, in a private space”, as this may be confusing for international readers may not know Swahili is a language.

We thank Reviewer #1 for this suggestion. We agree that the original wording may be unclear to readers unfamiliar with Swahili and have revised the sentence accordingly. The text now reads: “Interviews were conducted face-to-face in Swahili, in a private space, by Tanzanian research assistants.”

11. Reviewer #1: Please mention what quality assurance measures were taken, and if none were taken, why this was not done.

We thank Reviewer #1 for this comment. We have added additional detail to the Methods describing the procedures used throughout the coding and analysis process, including regular study team meetings to review coding decisions, refine the codebook, and ensure consistent application of codes across transcripts.

12. Reviewer #1: Under data analysis, page 9, line 188, the authors write: “Dominant themes were identified by respondent group and by the number of respondents reporting each theme”. I understand that different themes may have been dominant/relevant for different participant subgroups, but the number of respondents/participants reporting a theme appears to be quantifying the findings, which is not necessary for this type of work.

We thank Reviewer #1 for this thoughtful comment. We agree that the original wording may have implied that themes were identified solely on the basis of participant counts. While the prevalence of themes across interviews informed our assessment of thematic prominence, dominant themes were identified through iterative review of coded transcripts and consideration of their relevance to intervention acceptability. We have revised the text to clarify this point.

13. Reviewer #1: Related, please be consistent in the use of respondent or participant.

We thank Reviewer #1 for this suggestion. We agree and have revised the manuscript for consistency, replacing “respondent” with “participant” throughout.

14. Reviewer #1: Under ethical considerations, (page 9, line 197), I am missing some information. Typically, I would expect to read what happens to the audio files, what happens to the transcripts, what happens to the patient information, and how this is all done securely, where and how long data is stored for, etc.

We thank the reviewer for this suggestion. We have expanded the Ethical Considerations section to provide additional information regarding management of the qualitative data. Specifically, we now clarify that interviews were audio-recorded with participant consent, transcripts were de-identified prior to analysis, and study data were stored securely with access limited to authorized study personnel. We also note that data were managed in accordance with institutional and ethics committee requirements. We also specifically mention that study data will be stored on a secure server at KCMC for 10 years, as per Tanzania National Institute for Medical Research requirements.

15. Reviewer #1: If the interviewers/researchers had previous contacts with the healthcare providers (or patients) who were interviewed, I would appreciate this being mentioned briefly in the methods for the sake of transparency.

We thank the reviewer for this helpful suggestion. We have added additional detail to the Methods section describing the relationship between the research team and study participants. Specifically, we clarify that some members of the research team had prior professional relationships with KCMC ED staff participants, while interviews were conducted by Tanzanian research assistants with no prior supervisory, evaluative, or clinical relationship with any participant.

16. Reviewer #1: The themes are presented clear with supporting quotes in-text. I was wondering whether there were only themes, and if no sub-themes emerged during the process. I am typically used to a larger code-three and wonder if the data does not contain more in-depth information which is currently not provided as subthemes.

We thank Reviewer #1 for this observation. Our analysis was structured deductively around the seven domains of the TFA, which serve as the superordinate organizing framework, with 19 discrete themes identified within those domains. The TFA domains and themes therefore constitute a two-level hierarchy. Subthemes were not identified as a separate level in our analysis, as the 19 themes adequately captured the depth of the data within each TFA domain. We have added a clarifying sentence to the Data Analysis section to make this structure more explicit.

17. Reviewer #1: Page 29, line 522: the word transplant may be better replaced by ‘implement’ or another adjective.

We thank the reviewer for this comment. We have updated the manuscript wording accordingly.

18. Reviewer #1: As becomes clear in the results and discussion, some participants from this study were involved in the development of the intervention, and some of the authors work at the same institution as where the intervention was implemented, which may further lead to social desirability bias. Both are mentioned very briefly as a limitation, but I believe this is a large limitation that should be addressed more thoroughly, in particular how this may effected the findings, and what the implications of this may be.

We thank Reviewer #1 for this insightful comment. We have expanded the Limitations section to further discuss the potential for social desirability bias. Specifically, we discuss how participant involvement in the co-design process and investigator affiliations with the study institution may have contributed to more favorable perceptions of the intervention and the implications of this potential bias for interpretation of the findings.

19. Reviewer #1: On page 43, line 822, the authors write: “Additional study is needed to explore acceptability of the pamphlets in a broader patient population, including among patients who have limited literacy.” While I completely agree this is highly relevant, this is the first time literacy is mentioned. Do we know anything about the literacy or educational levels of the patients involved? Were the pamphlets not co-created with a group of individuals with varying levels of health literacy, since I would expect the authors to have done so.

We thank the reviewer for this helpful comment. As the reviewer notes, the patient educational pamphlet was developed through an iterative co-design process in which five patients recently admitted with AMI reviewed the educational materials and provided feedback regarding their comprehensibility and design. However, all 5 patient participants in the present study were literate. We have now clarified the literacy of our participants in the revised Results section. We have also revised the Discussion to clarify the co-design process while noting that additional study is needed to evaluate the acceptability of the pamphlet among patients with limited literacy, particularly those with no formal education.

20. Reviewer #1: I would remove this sentence: “Nonetheless, the consistency and specificity of responses support the credibility of the findings”, since this does not mitigate the limitations.

We thank the reviewer for this comment. We have removed the aforementioned sentence from the Limitations section.

21. Reviewer #1: I would appreciate a more critical discussion on the generalizability of the findings from this setting to other settings, including smaller or more rural hospitals, hospitals with more limited means, and otherwise different circumstances. Since this intervention was both co-created with individuals from this hospital, as well as implemented in this hospital, the generalizability may be a large limitation of the current study, but this is only briefly mentioned.

We thank Reviewer #1 for this helpful comment. We have expanded the Limitations section to further discuss the generalizability of these findings. Specifically, we note that MIMIC was co-designed and evaluated at a single tertiary referral hospital with an established emergency medicine program and that the generalizability of these findings may be limited, as intervention acceptability may differ in smaller, rural, or less-resourced facilities across Tanzania. We also discuss the need for future evaluation in diverse emergency care settings to determine whether adaptation of specific intervention components is needed.

22. Reviewer #1: Is there any quantitative data on improvements in effectiveness and efficiency? If so, I would appreciate this informatio

Attachment Submitted filename: R2R_R1_PLOS.docx

# Peer review: Decision Letter 1

**DOI:** 10.1371/journal.pone.0357564.r003

**Authors:** Buna Bhandari

20 Aug 2026

Stakeholder perceptions of the acceptability of an intervention to improve uptake of evidence-based emergency myocardial infarction care in Tanzania: A qualitative study

PONE-D-26-19842R1

Dear Dr. Bettger,

We’re pleased to inform you that your manuscript has been judged scientifically suitable for publication and will be formally accepted for publication once it meets all outstanding technical requirements.

Within one week, you’ll receive an e-mail detailing the required amendments. When these have been addressed, you’ll receive a formal acceptance letter and your manuscript will be scheduled for publication.

An invoice will be generated when your article is formally accepted. Please note, if your institution has a publishing partnership with PLOS and your article meets the relevant criteria, all or part of your publication costs will be covered. Please make sure your user information is up-to-date by logging into Editorial Manager at Editorial Manager® and clicking the ‘Update My Information' link at the top of the page. For questions related to billing, please contact billing support.

If your institution or institutions have a press office, please notify them about your upcoming paper to help maximize its impact. If they’ll be preparing press materials, please inform our press team as soon as possible -- no later than 48 hours after receiving the formal acceptance. Your manuscript will remain under strict press embargo until 2 pm Eastern Time on the date of publication. For more information, please contact onepress@plos.org.

Kind regards,

Dr Buna Bhandari

Academic Editor

PLOS One

Additional Editor Comments (optional):

Reviewers' comments:

Reviewer's Responses to Questions

**Comments to the Author**

Reviewer #1: All comments have been addressed

Reviewer #2: All comments have been addressed

**********

2. Is the manuscript technically sound, and do the data support the conclusions?

Reviewer #1: Yes

Reviewer #2: Yes

**********

3. Has the statistical analysis been performed appropriately and rigorously?

Reviewer #1: N/A

Reviewer #2: Yes

**********

4. Have the authors made all data underlying the findings in their manuscript fully available?

The PLOS Data policy

Reviewer #1: No

Reviewer #2: No

**********

5. Is the manuscript presented in an intelligible fashion and written in standard English?

Reviewer #1: Yes

Reviewer #2: Yes

**********

Reviewer #1: I want to congratulate the authors on this revised manuscript, as it has significantly improved due to the revisions. I feel the manuscript is ready to be accepted for publication.

Reviewer #2: Thank you to the authors for their thorough and satisfactory responses to all of the comments raised. The revisions and clarifications adequately address the concerns, and the manuscript has been substantially improved.

**********

what does this mean?). If published, this will include your full peer review and any attached files.

If you choose “no”, your identity will remain anonymous but your review may still be made public.

**Do you want your identity to be public for this peer review?**  For information about this choice, including consent withdrawal, please see our Privacy Policy

Reviewer #1: No

Reviewer #2: No

**********

# Editor report: Acceptance letter

**DOI:** 10.1371/journal.pone.0357564.r004

**Authors:** Buna Bhandari

PONE-D-26-19842R1

PLOS One

Dear Dr. Bettger,

I'm pleased to inform you that your manuscript has been deemed suitable for publication in PLOS One. Congratulations! Your manuscript is now being handed over to our production team.

At this stage, our production department will prepare your paper for publication. This includes ensuring the following:

* All references, tables, and figures are properly cited

* All relevant supporting information is included in the manuscript submission,

* There are no issues that prevent the paper from being properly typeset

You will receive further instructions from the production team, including instructions on how to review your proof when it is ready. Please keep in mind that we are working through a large volume of accepted articles, so please give us a few days to review your paper and let you know the next and final steps.

Lastly, if your institution or institutions have a press office, please let them know about your upcoming paper now to help maximize its impact. If they'll be preparing press materials, please inform our press team within the next 48 hours. Your manuscript will remain under strict press embargo until 2 pm Eastern Time on the date of publication. For more information, please contact onepress@plos.org.

You will receive an invoice from PLOS for your publication fee after your manuscript has reached the completed accept phase. If you receive an email requesting payment before acceptance or for any other service, this may be a phishing scheme. Learn how to identify phishing emails and protect your accounts at https://explore.plos.org/phishing.

If we can help with anything else, please email us at customercare@plos.org.

Thank you for submitting your work to PLOS One and supporting open access.

Kind regards,

PLOS One Editorial Office Staff

on behalf of

Dr. Buna Bhandari

Academic Editor

PLOS One
